# Low YTHDC1 Expression Upregulates FSCN1 to Promote Nuclear F‐Actin Formation and Facilitate Double‐strand DNA Breaks Repair in TMZ‐Resistant Glioblastoma

**DOI:** 10.1002/advs.202513632

**Published:** 2025-12-27

**Authors:** Minglong Yang, Wanxiang Niu, Yuanfei Wang, Peng Chen, Maolin Mu, Xiaoming Zhang, Ben Xu, Shanshan Hu, Chaoshi Niu, Pengfei Wu

**Affiliations:** ^1^ Department of Neurosurgery The First Affiliated Hospital of USTC Division of Life Sciences and Medicine University of Science and Technology of China Hefei Anhui China; ^2^ Anhui Key Laboratory of Brain Function and Diseases Hefei Anhui China; ^3^ Anhui Provincial Stereotactic Neurosurgical Institute Hefei Anhui China; ^4^ Anhui Provincial Clinical Research Center for Neurosurgical Disease Hefei Anhui China

**Keywords:** clinical therapy, DSBs repair, filamentous actin formation, glioblastoma, temozolomide resistance

## Abstract

Glioblastoma (GBM) is an aggressive and recurrent malignancy with a poor prognosis. Although temozolomide (TMZ) is a cornerstone of GBM treatment, its efficacy is often compromised by inherent or acquired resistance, underscoring the urgent need to uncover molecular mechanisms, discover new therapeutic targets, and develop innovative treatment strategies. In this study, we found an increased formation of filamentous actin (F‐actin) within the nuclei of TMZ‐resistant GBM cells. We also showed that overexpression of FSCN1 in TMZ‐resistant GBM cells promotes F‐actin formation and facilitates the repair of DNA double‐strand breaks (DSBs). Further investigation revealed a marked decrease in the expression of YTHDC1 in TMZ‐resistant GBM cells, which regulates FSCN1 through m6A modification. Additionally, FSCN1 activates the CDC42/N‐WASP/Arp2/3 signaling pathway by recruiting FGD1 to activate CDC42^GTP^, which drives nuclear F‐actin formation. Importantly, combining the FSCN1 inhibitor NP‐G2‐044, with TMZ therapy resulted in stronger anti‐tumor effects both in vitro and in vivo. In conclusion, the study demonstrates that nuclear F‐actin formation in GBM promotes DSB repair and reveals that targeting FSCN1 with NP‐G2‐044 could be a promising strategy for enhancing treatment outcomes and improving the prognosis for GBM patients.

## Graphical abstract

This study revealed that low expression of YTHDC1 in TMZ‐resistant GBM cells leads to increased FSCN1 expression, which is suppressed by m6A modification. FSCN1 activates the CDC42/N‐WASP/Arp2/3 axis in the cell nucleus by recruiting the guanine nucleotide exchange factor (GEF) family member FGD1, thereby promoting F‐actin polymerization in the nucleus. The study also identified the potential novel therapeutic strategy of combining TMZ with NP‐G2‐044. These findings provide theoretical support for combined therapies in the clinical treatment of GBM resistance.

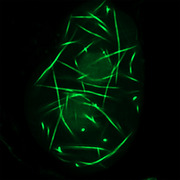

## 1 Introduction

Glioblastoma (GBM) is the most common and aggressive primary brain tumor [1, 2, 3], with a 5‐year survival rate of just 7.2% [4]. The current standard treatment involves surgical resection followed by radiation and adjuvant chemotherapy with temozolomide (TMZ) [5, 6, 7]. Despite extensive research, effective treatment options for GBM remain limited. Resistance to chemotherapy and radiation is linked to increased DNA damage response (DDR) signaling and enhanced tumor cell survival [8, 9, 10, 11], which are further promoted by disrupted oncogenic and epigenetic pathways. Identifying new mechanisms that regulate DDR in GBM could lead to new therapeutic strategies to slow tumor growth, reduce recurrence, and ultimately improve patient outcomes [12, 13, 14]. However, the role of F‐actin in chemotherapy resistance in GBM remains underexplored. Therefore, understanding how nuclear F‐actin contributes to TMZ resistance in GBM cells is crucial for developing targeted therapies.

The actin cytoskeleton plays a crucial role in regulating cell shape, motility, and cargo transport [15], primarily through the controlled polymerization of globular actin (G‐actin) into F‐actin [15]. While actin is predominantly found in the cytoplasm, F‐actin structures also occur in the nucleus, where they participate in processes such as mitotic exit [16], serum response [17], and homology‐directed DNA double‐strand break (DSB) repair [18, 19, 20, 21]. In Drosophila and mouse cells, where heterochromatin forms distinct nuclear domains known as chromocenters in mice, DSB recognition and resection initially occur within these domains [22, 23, 24]. Over time, the heterochromatin domain expands, and DSBs are relocated to regions outside the domain [23, 25, 26]. This relocation is facilitated by a network of nuclear actin filaments that are assembled at the repair sites by the Arp2/3 complex and extend toward the nuclear periphery [18, 19]. Recent studies have shown that FSCN1 is essential for the formation of endogenous nuclear actin bundles [27]. These bundlers are transported into the nucleus, where they support post‐mitotic F‐actin bundling, enhance the DNA damage response [27, 28], and contribute to cancer cell survival [29, 30, 31]. Our findings suggest that elevated FSCN1 expression levels promote nuclear F‐actin formation, which contributes to resistance to TMZ in GBM.

m6A is the most common and abundant internal modification of mRNA in eukaryotes, playing a key role in regulating gene expression. It exerts its effects through three main components: “WRITERS” “ERASERS” and “READERS”. This modification is crucial in controlling various cellular processes [32]. YTHDC1, one of the key m6A “readers” has been shown to influence RNA stability and decay by binding to m6A‐marked mRNAs [33, 34]. Moreover, recent studies have linked alterations in YTHDC1 expression to tumor progression and resistance [35, 36, 37, 38]. However, the role of YTHDC1 in TMZ‐resistant GBM and its underlying mechanism remain unexplored.

In this study, we show that low expression of YTHDC1 in GBM leads to increased FSCN1 expression through m6A modification. FSCN1 then regulates nuclear F‐actin formation by recruiting FGD1 to activate the CDC42/N‐WASP/Arp2/3 pathway. This process enhances DNA double‐strand break repair, contributing to TMZ resistance. Additionally, we demonstrate that the FSCN1 inhibitor NP‐G2‐044, currently in clinical trials, combined with TMZ, significantly improves the therapeutic effect in GBM, both in vitro and in vivo.

## 2 Results

### 2.1 Nuclear F‐Actin Increase Promotes TMZ Resistance in GBM Cells

**FIGURE 1:**
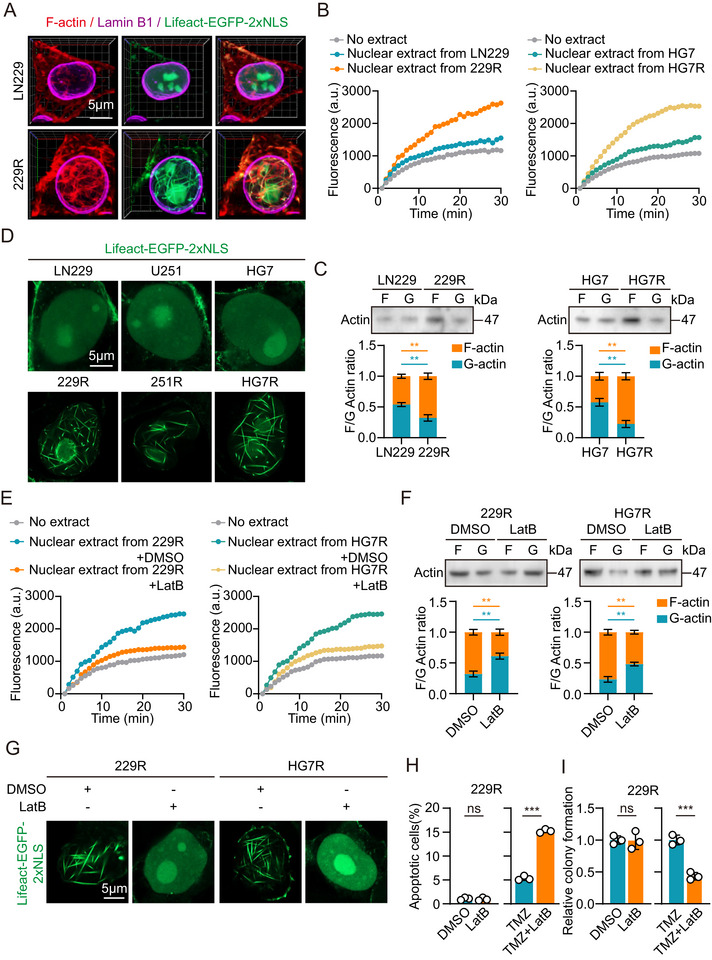
Nuclear F‐actin increase and promote TMZ resistance in GBM cells. (A) Immunofluorescence analysis of GBM cells stained with phalloidin, anti‐Lamin B1 antibody and Lifeact‐EGFP‐2xNLS plasmids. Scale bar = 5µm. (B) Normalized timecourse of pyrene‐labelled actin assembly in the LN229, 229R, HG7 and HG7R nuclear extracts. (C) Western Blot (top) and analysis of globular (G‐) and filamentous (F‐) actin indicate the rate of actin polymerization in GBM cells (n = 3). (D) Immunofluorescence analysis of GBM cells treated with Lifeact‐EGFP‐2xNLS plasmids. Scale bar = 5µm. (E) Normalized timecourse of pyrene‐labelled actin assembly in the TMZ‐resistant GBM cells nuclear extracts after DMSO or LatB treatment. (F) Western Blot (top) and analysis of globular (G‐) and filamentous (F‐) actin indicate the rate of actin polymerization in TMZ‐resistant cells after DMSO or LatB treatment (n = 3). (G) Immunofluorescence analysis of TMZ‐resistant GBM cells treated with Lifeact‐EGFP‐2xNLS plasmids after DMSO or LatB treatment. Scale bar = 5µm. (H) Flow cytometric analysis revealed the effect of F‐actin suppressed on the apoptosis of TMZ‐resistant cells with or without TMZ treatment (n = 3). (I) Colony formation assay detected the effect of F‐actin suppressed on the growth of TMZ‐resistant cells with or without TMZ treatment in a 6‐well dish (800 cells per well) for 11 days (n = 3). Representative images and the relative number of colonies are shown. For A, D and G, scale bars, 5 µm. Data were analyzed using Student's t‐test (C, F, H and I). Significant results were presented as NS non‐significant, *p < 0.05, **p < 0.01, ***p < 0.001.

The changes in the actin cytoskeleton of tumor cells impact their behavior and biological functions. To investigate the alterations of actin in the nuclei of TMZ‐resistant GBM cells, we performed immunofluorescence staining on GBM cell lines (LN229, U251, HG7) and their corresponding TMZ‐resistant GBM cell lines (229R, 251R, HG7R) using Lifeact and a GFP‐tagged plasmid with a nuclear localization signal (NLS) (Lifeact‐EGFP‐2xNLS). The 3D view results showed that the nuclear F‐actin in TMZ‐resistant GBM cells was increased compared to parental GBM cells, and the Lifeact‐EGFP‐2xNLS staining exhibited better specificity than phalloidin staining (Figure 1A; Figure S1A). We quantified the proportion of cells with nuclear F‐actin positivity through immunofluorescence staining. The results showed a significant increase in the proportion of cells with nuclear F‐actin positivity in TMZ‐resistant GBM cells (Figure S1B). Subsequently, pyrene‐actin assays demonstrated that nuclear extracts from TMZ‐resistant GBM cells promoted more F‐actin polymerization than those from parental GBM cells (Figure 1B; Figure S1C,D). F‐actin/G‐actin assays revealed that the F/G actin ratio in nuclear extracts from TMZ‐resistant GBM cells was also higher than that from parental GBM cells [39] (Figure 1C; Figure S1E). To confirm these findings, we visualized endogenous nuclear actin using Lifeact‐EGFP‐2xNLS and imaged it using the Airyscan mode of a confocal microscope. We observed a significant increase in nuclear F‐actin in TMZ‐resistant GBM cells [16] (Figure 1D).

Next, we aimed to investigate the role of nuclear F‐actin in TMZ resistance. To inhibit nuclear actin polymerization, TMZ‐resistant GBM cells were pretreated with 1 µM Latrunculin B (LatB) for 30 min. This treatment significantly reduced the formation of nuclear F‐actin in TMZ‐resistant GBM cells compared to the DMSO‐treated group (Figure 1E–G; Figure S1F–I). Additionally, to explore the relationship between F‐actin and TMZ resistance, we treated the cells with TMZ after inhibiting F‐actin formation, followed by flow cytometry and colony formation assays. The results showed that LatB inhibition of nuclear actin polymerization had no significant impact on apoptosis or proliferation in TMZ‐resistant GBM cells. However, TMZ‐resistant GBM cells treated with both TMZ and LatB showed increased apoptosis and decreased colony formation compared to those treated with TMZ alone (Figure 1H,I; Figure S1J,K). These results suggest that the formation and function of nuclear F‐actin play a critical role in the development of TMZ resistance in GBM cells.

### 2.2 FSCN1 Regulates Nuclear F‐Actin Formation to Promote DNA Damage Repair

**FIGURE 2:**
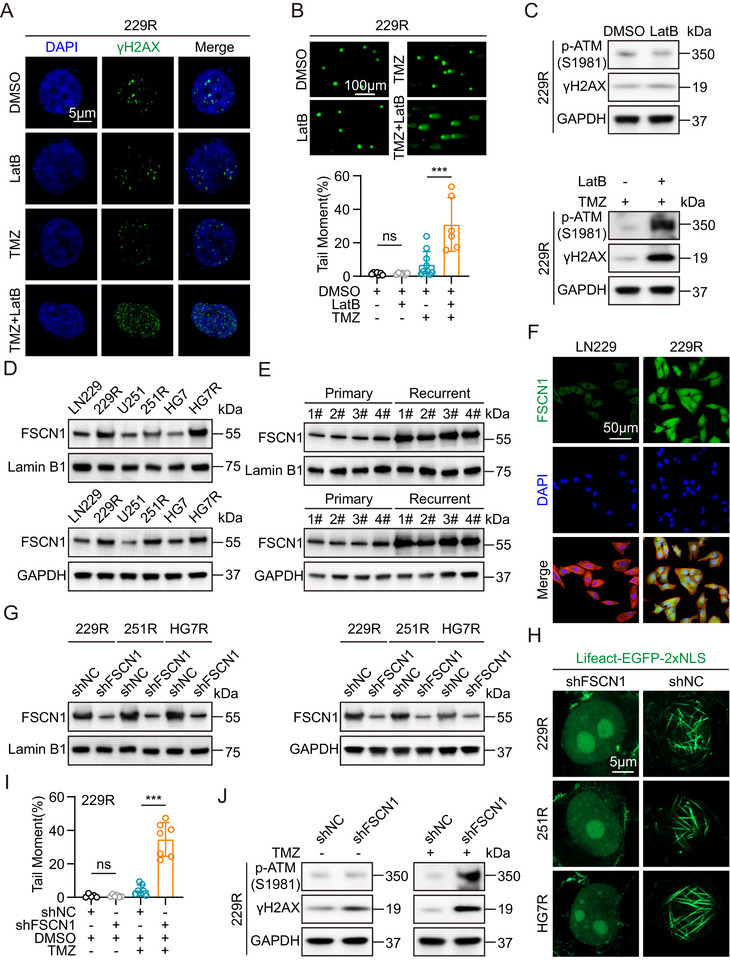
FSCN1 regulates nuclear F‐actin formation to promote DNA damage repair. (A) Immunofluorescence analysis of γH2AX in TMZ‐resistant GBM cells treated with DMSO, TMZ or LatB. The nuclei were stained with DAPI. Quantification of γH2AX in Image J. Scale bar = 5µm. (B) Comet assays measuring the DNA damage degree in 229R cells treated with DMSO, LatB, TMZ and TMZ + LatB. Scale bar = 100µm. Comet tails were measured by image j. (C) Western blot analysis of p‐ATM and γH2AX expression in TMZ‐resistant GBM cells treated with DMSO, TMZ or LatB. (D) Western blot analysis of FSCN1 expression in parental and TMZ‐resistant GBM cell nucleus (top) and western blot analysis of FSCN1 expression in parental and TMZ‐resistant GBM cell (bottom). (E) Western blot analysis of FSCN1 expression in paired primary and recurrent tissue cell nucleus from clinical patients (top) and western blot analysis of FSCN1 expression in paired primary and recurrent tissue cell from clinical patients (bottom). (F) Immunofluorescence analysis of FSCN1 expression in parental and TMZ‐resistant GBM cells. Scale bar = 50µm. (G) Western blot analysis of FSCN1 expression in FSCN1 knockdown TMZ‐resistant GBM cell nucleus (left) and western blot analysis of FSCN1 expression in FSCN1 knockdown TMZ‐resistant GBM cells (right). (H) Immunofluorescence analysis of FSCN1 knockdown TMZ‐resistant GBM cells treated with Lifeact‐EGFP‐2xNLS plasmids. Scale bar = 5µm. (I) Comet assays measuring the DNA damage degree in shNC 229R cells treated with DMSO, TMZ and shFSCN1 229R cells treated with DMSO and TMZ. (J) Western blot analysis of p‐ATM and γH2AX expression in shFSCN1 229R cells treated with or without TMZ. For A, and H, scale bars, 5 µm. For F, scale bars, 50 µm. For B, scale bars, 100 µm. Data were analyzed using Student's t‐test (B and I). Significant results were presented as NS non‐significant, *p < 0.05, **p < 0.01, ***p < 0.001.

Previous studies have shown that nuclear F‐actin is associated with the repair of DNA double‐strand breaks (DSBs). To investigate the relationship between nuclear F‐actin and DNA damage repair in GBM cells, we treated TMZ‐resistant GBM cells with DMSO, LatB, TMZ, and TMZ+LatB. We assessed nuclear DSB damage using immunofluorescence, comet assay, and Western blot. The results showed that LatB treatment alone did not induce significant changes in DNA damage in TMZ‐resistant GBM cells. However, combined TMZ and LatB treatment resulted in an increase in nuclear DNA damage sites, DNA damage tails, and the accumulation of DSB‐associated marker proteins compared to TMZ treatment alone (Figure 2A–C; Figure S2A–C) [11, 40, 41, 42]. Next, we aimed to identify key molecules that influence F‐actin polymerization in TMZ‐resistant GBM cells. After preliminary experiments with various actin‐regulating proteins, we focused on the actin‐bundling protein fascin (FSCN1). We then performed Western blot and immunofluorescence experiments to examine the levels of FSCN1 in the nucleus and total cell extracts of both parental and TMZ‐resistant GBM cells. The results showed that FSCN1 expression was significantly elevated in the nuclei and total protein levels of TMZ‐resistant GBM cells, as well as in tissues from recurrent GBM patients (Figure 2D–F; Figure S2D). Furthermore, qRT‐PCR results revealed that FSCN1 mRNA expression was significantly increased in TMZ‐resistant GBM cells and recurrent GBM patient tissues compared to parental GBM cells and primary GBM patient tissues (Figure S2E,F). To study the relationship between FSCN1 expression levels and nuclear F‐actin formation in GBM cells, we generated shFSCN1 cell models in TMZ‐resistant GBM cells. We confirmed FSCN1 expression through qRT‐PCR and Western blot, and the results showed that FSCN1 protein expression in both the nucleus and total cell extracts was reduced in shFSCN1‐treated TMZ‐resistant GBM cells, and FSCN1 mRNA expression levels were also lower (Figure 2G; Figure S2G). Immunofluorescence staining of nuclear F‐actin revealed that nuclear F‐actin formation was suppressed in shFSCN1‐treated TMZ‐resistant GBM cells (Figure 2H). To further explore the functional role of FSCN1, we analyzed data from the TCGA dataset and classified patients into high and low FSCN1 expression groups. Gene Ontology (GO) analysis indicated that FSCN1 expression was associated with DNA double‐strand break repair and the formation of DNA damage repair complexes (Figure S3A). Subsequently, we assessed DNA damage levels after shFSCN1 treatment using comet assay and Western blot. The results showed that shFSCN1 significantly increased DNA damage tails and the expression of DNA damage‐related marker proteins in TMZ‐resistant GBM cells following TMZ treatment (Figure 2I,J; Figure S3B,C).

### 2.3 YTHDC1 Regulates FSCN1 Expression Through m6A Modification

**FIGURE 3:**
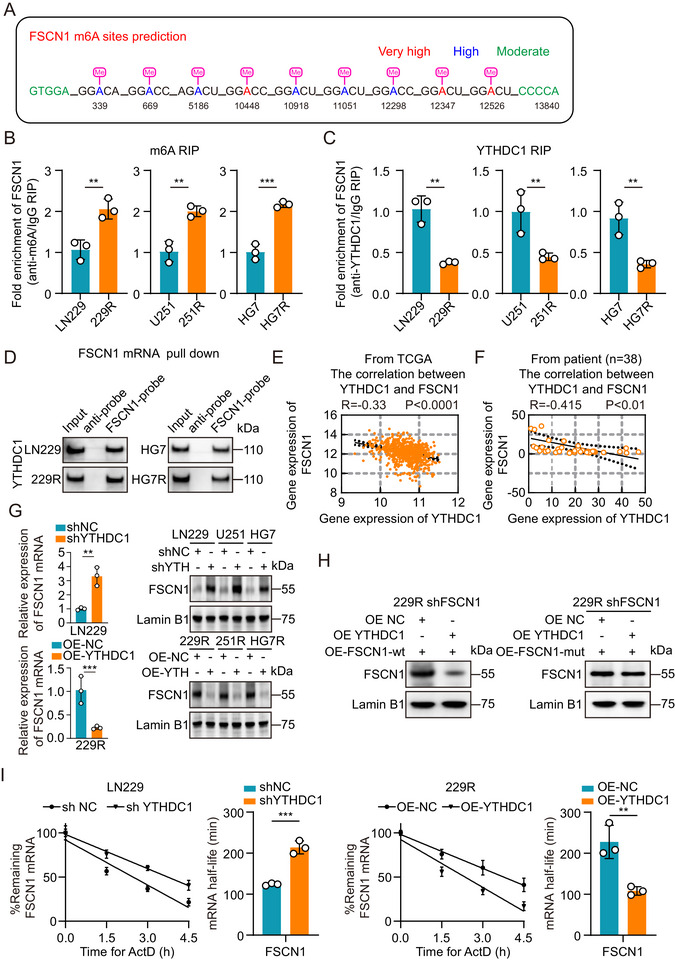
YTHDC1 regulates FSCN1 expression through m6A modification. (A) Prediction of m6A modification sites of FSCN1 mRNA. (B) RIP‐qPCR assay of FSCN1 mRNA with m6A antibody normalized to IgG in parental and TMZ‐resistant GBM cells (n = 3). (C) RIP‐qPCR assay of FSCN1 mRNA with YTHDC1 antibody normalized to IgG in parental and TMZ‐resistant GBM cells (n = 3). (D) Western blot analysis of YTHDC1 after pulldown by FSCN1 mRNA probes in parental and TMZ‐resistant GBM cells. (E) Pearson correlation analysis between the YTHDC1 and FSCN1 levels in GBM sample tissues from TCGA dataset. (F) Pearson correlation analysis between theYTHDC1 and FSCN1 levels in GBM sample tissues (n = 38). (G) Relative expression of FSCN1 mRNA in YTHDC1 knockdown parental GBM cells and YTHDC1 overexpression TMZ‐resistant GBM cells (left, n = 3). Western blot analysis of FSCN1 in YTHDC1 knockdown parental and YTHDC1 overexpression TMZ‐resistant GBM cells (right). (H) Western blot analysis of nuclear FSCN1 after OE‐YTHDC1 in 229R shFSCN1 OE‐FSCN1‐wt cells and 229R shFSCN1 OE‐FSCN1‐mut cells. (I) Relative expression of FSCN1 mRNA in YTHDC1 knockdown LN229 and YTHDC1 overexpression 229R after treatment with 80 nM Actinomycin D for 0h, 1.5h, 3h and 4.5h (n = 3). Data were analyzed using Student's t‐test (B, C, G and I) and Pearson correlation coefficient (E and F). Significant results were presented as NS non‐significant, *p < 0.05, **p < 0.01, ***p < 0.001.

Building on our previous research, we used the online tool SRAMP to predict multiple high‐confidence and very high‐confidence m6A binding sites on FSCN1 mRNA (Figure 3A). To validate these predictions, we performed RNA immunoprecipitation (RIP) experiments in GBM cells, using an m6A antibody and magnetic beads to capture m6A‐bound mRNA. After purification, we detected the pulled‐down FSCN1 mRNA in GBM cells through qRT‐PCR (Figure 3B). To identify the m6A methyltransferases involved in FSCN1 regulation, we analyzed previous sequencing datasets and m6A enzyme profiles. The results showed that YTHDC1 was the only common factor (Figure S4A). Our previous sequencing data revealed that YTHDC1 was consistently downregulated in TMZ‐resistant GBM cells and recurrent GBM patient samples (Figure S4B). We then investigated the interaction between YTHDC1 protein and FSCN1 mRNA through RIP and pull‐down experiments. The results showed that YTHDC1 protein interacts with FSCN1 mRNA in GBM cells (Figure 3C,D; Figure S4C). Correlation analysis of YTHDC1 and FSCN1 expression in public databases (TCGA) and clinical GBM patient samples showed a significant negative correlation between YTHDC1 and FSCN1 expression (Figure 3E,F). To further explore the effect of YTHDC1 protein on FSCN1 expression, we established s∖ shYTHDC1 cell lines in parental GBM cells (LN229, U251, HG7) and stable OE‐YTHDC1 cell lines in TMZ‐resistant GBM cells (229R, 251R, HG7R), and verified YTHDC1 expression levels through qRT‐PCR and Western blot experiments (Figure S4D,E). We then examined FSCN1 expression levels through qRT‐PCR and Western blot, and the results showed that in parental GBM cells, shYTHDC1 led to increased FSCN1 expression, whereas in TMZ‐resistant GBM cells, OE‐YTHDC1 suppressed FSCN1 expression (Figure 3G; Figure S4F,G). To further validate the specificity of the interaction between YTHDC1 protein and FSCN1 mRNA, we mutated two highly confident m6A binding sites on FSCN1 mRNA. After overexpressing FSCN1‐wt and FSCN1‐mut in 229R shFSCN1 cells, we again overexpressed YTHDC1. Western blot results showed that in 229R shFSCN1 cells, OE‐FSCN1‐wt followed by OE‐YTHDC1 led to a decrease in nuclear FSCN1 protein, while OE‐FSCN1‐mut followed by OE‐YTHDC1 showed no significant change in nuclear FSCN1 (Figure 3H). We then used Actinomycin D to inhibit transcription and measured FSCN1 mRNA expression levels in LN229 shYTHDC1 cells and 229R OE‐YTHDC1 cells at 0, 1.5, 3, and 4.5 h through qRT‐PCR. The results showed that with reduced YTHDC1 expression, the decay rate of FSCN1 mRNA was significantly reduced (Figure 3I).

### 2.4 YTHDC1 Downregulation in TMZ‐Resistant GBM Cells and Recurrent Patients Correlates with TMZ Resistance

**FIGURE 4:**
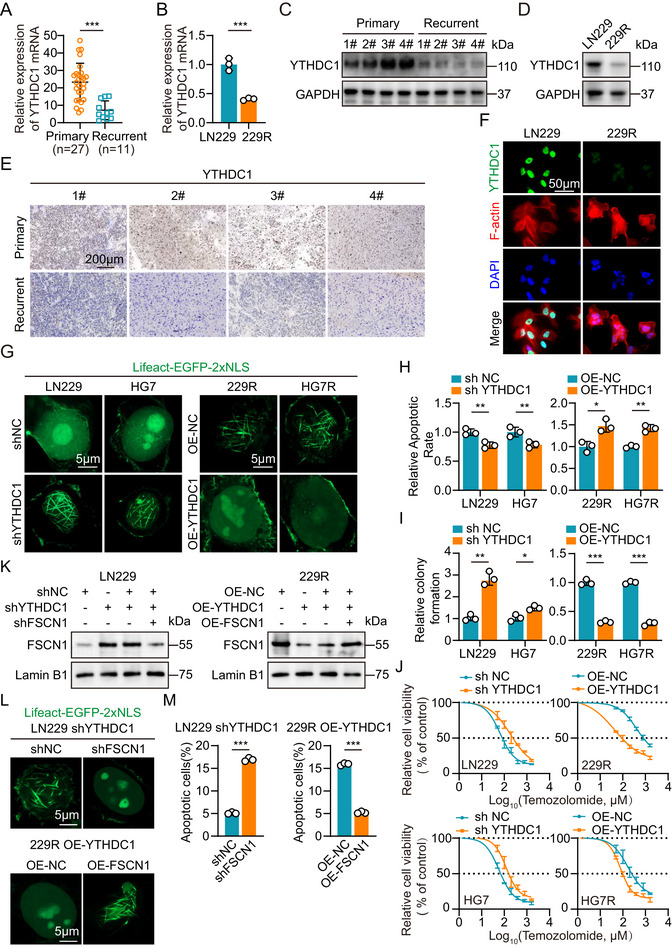
YTHDC1 downregulation in TMZ‐resistant GBM cells and recurrent patients correlates with TMZ resistance. (A) Relative expression of YTHDC1 in primary (n = 27) and recurrent (n = 11) GBM sample tissues. (B) Relative expression of YTHDC1 mRNA in parental and TMZ‐resistant GBM cells (n = 3). (C) Western blot analysis of YTHDC1 expression in paired primary and recurrent tissues from clinical patients. (D) Western blot analysis of YTHDC1 expression in parental and TMZ‐resistant GBM cells. (E) IHC staining of YTHDC1 in paired primary and recurrent tissues from clinical patients. Scale bar = 200µm. (F) Immunofluorescence analysis of YTHDC1 expression in parental and TMZ‐resistant GBM cells. The nuclei were stained with DAPI. Cytoskeletal staining with phalloidin. Scale bar = 50µm. (G) Immunofluorescence analysis of YTHDC1 expression in GBM cells treated with Lifeact‐EGFP‐2xNLS plasmids. Scale bar = 5µm. (H) Flow cytometric analysis revealed the apoptosis of YTHDC1 knockdown parental GBM cells and YTHDC1 overexpression TMZ‐resistant GBM cells with TMZ treatment (n = 3). (I) Colony formation assay detected the growth of YTHDC1 knockdown parental GBM cells and YTHDC1 overexpression TMZ‐resistant GBM cells with TMZ treatment in a 6‐well dish (800 cells per well) for 11 days (n = 3). (J) CCK‐8 assay analysis revealed the effect of YTHDC1 knockdown in parental GBM cells and YTHDC1 overexpression TMZ‐resistant GBM cells with TMZ treatment at the indicated concentrations for 72 h (n = 3). (K) Western blot analysis of FSCN1 rescue experiment in LN229 shYTHDC1 cells with shFSCN1 and in 229R OE‐YTHDC1 cells with OE‐FSCN1. (L) Immunofluorescence with Lifeact‐EGFP‐2xNLS plasmids treatment analysis of FSCN1 rescue experiment in LN229 shYTHDC1 cells with shFSCN1 and in 229R OE‐YTHDC1 cells with OE‐FSCN1. Scale bar = 5µm. (M) Flow cytometric analysis of FSCN1 rescue experiment in LN229 shYTHDC1 cells with shFSCN1 and in 229R OE‐YTHDC1 cells with OE‐FSCN1 (n = 3). For G and L, scale bars, 5 µm. For F, scale bars, 50 µm. For E, scale bars, 200 µm. Data were analyzed using Student's t‐test (A, B, H, I and M) and four‐parameter logistic regression (J). Significant results were presented as NS non‐significant, *p < 0.05, **p < 0.01, ***p < 0.001.

To further investigate YTHDC1 expression in GBM tissues and cells, we examined the mRNA levels of YTHDC1 in tumor samples from 27 primary and 11 recurrent GBM patients using qRT‐PCR. The results showed that YTHDC1 expression was significantly reduced in recurrent GBM tissues, and consistent results were observed in four paired samples (primary vs. recurrent GBM tissues) (Figure 4A; Figure S5A). Moreover, qRT‐PCR analysis revealed that YTHDC1 expression levels were markedly decreased in TMZ‐resistant GBM cells compared to their parental counterparts (Figure 4B; Figure S5B). Western blot results further confirmed that YTHDC1 protein levels were significantly lower in both recurrent GBM tissues and TMZ‐resistant GBM cells compared to primary tumors and parental cells (Figure 4C,D; Figure S5C). Immunohistochemistry (IHC) staining also demonstrated a marked reduction of YTHDC1 protein levels in recurrent GBM tumors (Figure 4E), while immunofluorescence staining showed a significant decrease in YTHDC1 protein levels in TMZ‐resistant GBM cells (Figure 4F; Figure S5D). To explore the relationship between YTHDC1 expression and TMZ resistance, we analyzed patient data from the TCGA database, revealing that lower YTHDC1 expression was associated with poorer survival outcomes (Figure S5E). Subsequently, immunofluorescence staining of nuclear F‐actin in GBM cells showed that shYTHDC1 in parental GBM cells promoted nuclear F‐actin formation, whereas OE‐YTHDC1 in TMZ‐resistant GBM cells suppressed nuclear F‐actin filament formation (Figure 4G; Figure S5F). To determine the functional role of YTHDC1 in TMZ resistance, we performed flow cytometry apoptosis assays, colony formation assays, and CCK8 assays. The results showed that in parental GBM cells, shYTHDC1 reduced apoptosis and enhanced proliferation under TMZ treatment (Figure 4H–J; Figure S5G–I). Next, we performed rescue experiments by knocking down FSCN1 in LN229 shYTHDC1 cells and overexpressing FSCN1 in 229R OE‐YTHDC1 cells (Figure 4K). Immunofluorescence staining revealed that shFSCN1 inhibited nuclear F‐actin formation in LN229 shYTHDC1 cells, whereas OE‐FSCN1 restored nuclear F‐actin formation in 229R OE‐YTHDC1 cells (Figure 4L). After TMZ treatment, flow cytometry apoptosis assays showed that shFSCN1 restored TMZ sensitivity in LN229 shYTHDC1 cells, while OE‐FSCN1 reinstated TMZ resistance in 229R OE‐YTHDC1 cells (Figure 4M).

### 2.5 FSCN1 Activates the CDC42/N‐WASP/Arp2/3 Axis by Recruiting FGD1

**FIGURE 5:**
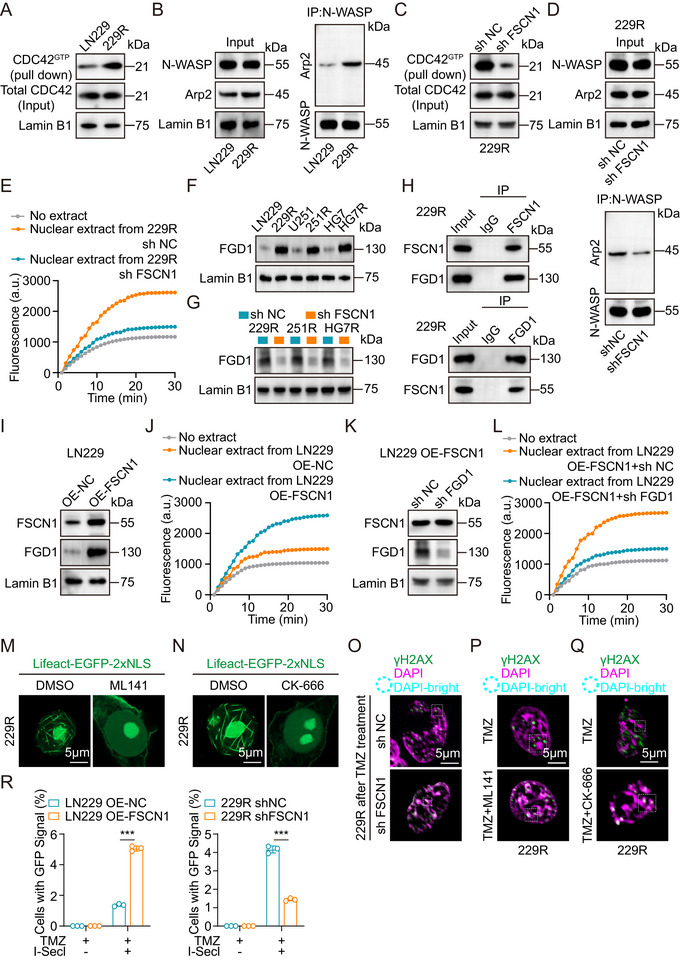
FSCN1 activates the CDC42/N‐WASP/Arp2/3 axis by recruiting FGD1. (A) Western blot analysis of CDC42 after pulldown by PAK‐PBD in the nucleus of LN229 and 229R cells. (B) Left: Western blot analysis in the nucleus of LN229 and 229R GBM cells with N‐WASP antibody, Arp2 antibody and Lamin B1 antibody. Right: Immunoprecipitation in the nucleus of LN229 and 229R GBM cells with an antibody against N‐WASP; the cells were subsequently labeled with the anti‐Arp2 antibody. (C) Western blot analysis of CDC42 after pulldown by PAK‐PBD in the nucleus of 229R shNC and 229R shFSCN1 GBM cells. (D) Upper: Western blot analysis in the nucleus of 229R shNC and 229R shFSCN1 GBM cells with N‐WASP antibody, Arp2 antibody and Lamin B1 antibody. Lower: Immunoprecipitation in the nucleus of 229R shNC and 229R shFSCN1 cells with an antibody against N‐WASP; the cells were subsequently labeled with the anti‐Arp2 antibody. (E) Normalized timecourse of pyrene‐labelled actin assembly in 229R shNC and 229R shFSCN1 cell nuclear extracts. (F) Western blot analysis of FGD1 in parental and TMZ‐resistant GBM cell nuclear extracts. (G) Western blot analysis of FGD1 in 229R shNC and 229R shFSCN1 GBM cell nuclear extracts. (H) Upper: Immunoprecipitation of 229R GBM cells with an antibody against FSCN1; the cells were subsequently labeled with the anti‐FGD1 antibody. Lower: Immunoprecipitation of 229R GBM cells with an antibody against FGD1; the cells were subsequently labeled with the anti‐FSCN1 antibody. (I) Western blot analysis in the nucleus of LN229 OE‐NC and LN229 OE‐FSCN1 GBM cells with FSCN1 antibody, FGD1 antibody and Lamin B1 antibody. (J) Normalized timecourse of pyrene‐labelled actin assembly in LN229 OE‐NC and LN229 OE‐FSCN1 GBM cell nuclear extracts. (K) Western blot analysis in the nucleus of LN229 OE‐FSCN1 shNC and LN229 OE‐FSCN1 shFGD1 GBM cells with FSCN1 antibody, FGD1 antibody and Lamin B1 antibody. (L) Normalized timecourse of pyrene‐labelled actin assembly in LN229 OE‐FSCN1 shNC and LN229 OE‐FSCN1 shFGD1 GBM cell nuclear extracts. (M) Immunofluorescence analysis of 229R cells treated with Lifeact‐EGFP‐2xNLS plasmids after treatment with DMSO or ML141 (10 µM, 37°C, 1 h). Scale bar = 5µm. (N) Immunofluorescence analysis of 229R cells treated with Lifeact‐EGFP‐2xNLS plasmids after treatment with DMSO or CK‐666 (100 µM, 37°C, 6 h). Scale bar = 5µm. (O) Immunofluorescence of γH2AX foci in FSCN1 knockdown TMZ‐resistant GBM cells with TMZ treatment show γH2AX foci in DAPIbright heterochromatin. Scale bar = 5µm. (P) Immunofluorescence of γH2AX foci in TMZ‐resistant GBM cells with TMZ or TMZ + ML141 (10 µM, 37°C, 1 h) treatment show γH2AX foci in DAPIbright heterochromatin. Scale bar = 5µm. (Q) Immunofluorescence of γH2AX foci in TMZ‐resistant GBM cells with TMZ or TMZ + CK‐666 (100 µM, 37°C, 6 h) treatment show γH2AX foci in DAPIbright heterochromatin. Scale bar = 5µm. (R) Detection of HR activity using the DR‐GFP reporter system. Different combinations of DR‐GFP reporter plasmids were co‐transfected into LN229 shNC and shFSCN1 cells, as well as 229R OE‐NC and OE‐FSCN1 cells. Cells were treated with 100µM TMZ for 24 h, and the percentage of GFP‐positive cells was determined by flow cytometry (n = 3). For M, N, O, P and Q scale bars, 5µm. Data were analyzed using Student's t‐test (R). Significant results were presented as NS non‐significant, *p < 0.05, **p < 0.01, ***p < 0.001.

To investigate the mechanism by which FSCN1 regulates actin filament formation and DNA double‐strand break (DSB) repair, we divided GBM patients into high and low FSCN1 expression groups based on gene set data from the TCGA database. KEGG pathway enrichment analysis showed that the “actin cytoskeleton regulation” pathway was significantly associated with our experimental results (Figure S6A). Further gene set enrichment analysis (GSEA) also confirmed that FSCN1 expression could activate the “actin cytoskeleton regulation” pathway (Figure S6B). Building on previous research, we found that FSCN1 expression might be closely linked to the activation of the CDC42/N‐WASP/Arp2/3 signaling axis [43, 44, 45, 46, 47, 48, 49]. Next, we assessed CDC42 activation levels (CDC42^GTP^) in LN229 and 229R cells through Western blot. The results showed that while the total CDC42 levels in the nuclear extracts of 229R cells did not show significant changes, CDC42^GTP^ levels were markedly elevated (Figure 5A). Comparison of N‐WASP and Arp2/3 levels in the nuclear extracts of LN229 and 229R cells revealed no significant difference in their nuclear protein levels. However, through immunoprecipitation (IP) with an N‐WASP antibody and quantitative analysis of Arp2, we found that more Arp2 protein was bound to N‐WASP in the nuclei of 229R cells (Figure 5B). These results suggest that the CDC42/N‐WASP/Arp2/3 pathway is activated in TMZ‐resistant GBM cells. We then investigated the activation of this pathway in the nuclear extracts of 229R shFSCN1 cells. Western blot and pyrene‐actin assays showed that shFSCN1 significantly inhibited CDC42 activation and the binding of N‐WASP and Arp2/3 (Figure 5C–E). Since FSCN1 itself does not directly activate CDC42, we performed FSCN1 immunoprecipitation and mass spectrometry analysis to identify its binding partners. The screening results showed that the GEF family member FGD1 could bind to FSCN1 and activate CDC42. Western blot analysis revealed that FGD1 expression was elevated in the nuclei of TMZ‐resistant GBM cells, and that shFSCN1 significantly reduced FGD1 protein levels (Figure 5F,G). IP experiments further confirmed the interaction between FSCN1 and FGD1 (Figure 5H). Next, we overexpressed FSCN1 in LN229 cells (OE‐FSCN1) and examined the levels of FGD1 and the activation of the CDC42/N‐WASP/Arp2/3 pathway through Western blot and pyrene‐actin assays. The results showed that OE‐FSCN1 increased FGD1 protein levels in the nucleus and activated the CDC42/N‐WASP/Arp2/3 pathway (Figure 5I,J). We then performed rescue experiments by knocking down FGD1 in LN229 OE‐FSCN1 cells. Western blot and pyrene‐actin assays revealed that shFGD1 suppressed nuclear F‐actin formation in LN229 OE‐FSCN1 cells (Figure 5K,L), suggesting that FSCN1 cannot independently activate the CDC42/N‐WASP/Arp2/3 pathway without FGD1. To explore the therapeutic potential of the CDC42/N‐WASP/Arp2/3 pathway, we used small‐molecule inhibitors of CDC42 (ML141) and Arp2/3 (CK‐666). Immunofluorescence results showed that inhibition of either CDC42 or Arp2/3 blocked F‐actin formation in TMZ‐resistant GBM cells (Figure 5M,N; Figure S6C,D). Moreover, using genetic methods to knock down CDC42, N‐WASP, and Arp2 in 229R cells, we assessed F‐actin formation and DNA damage through Western blot, pyrene‐actin assays, and comet assays. The results showed that shCDC42, shN‐WASP, and shARP2 significantly inhibited nuclear F‐actin polymerization and increased DNA damage tailing after TMZ treatment (Figure S6E–G). Finally, after treatment with shFSCN1 or the inhibitors ML141 and CK‐666 in TMZ‐resistant GBM cells, immunofluorescence analysis revealed that DNA double‐strand break (DSB) repair sites failed to migrate from heterochromatin regions to their periphery for homologous recombination (HR) repair after TMZ treatment [19] (Figure 5O–Q; Figure S6H–J). To investigate the relationship between FSCN1 expression levels and the frequency of HR repair events within cells, we used the DR‐GFP system in LN229 OE‐NC, OE‐FSCN1, and 229RshNC and shFSCN1 cells. Following TMZ treatment with or without ISceI expression, we performed flow cytometry to quantify the percentage of GFP‐positive cells. The results showed that in GBM cells, higher FSCN1 expression led to a higher frequency of HR repair under TMZ treatment (Figure 5R). In summary, these results suggest that FSCN1 promotes DSB repair by recruiting FGD1 to activate the CDC42/N‐WASP/Arp2/3 signaling axis, facilitating the migration of DSB repair sites from heterochromatin regions to their periphery for homologous recombination repair in GBM cells.

### 2.6 Treatment with TMZ Combined with the FSCN1 Inhibitor NP‐G2‐044 Reverses GBM TMZ Resistance Both In Vitro and In Vivo

**FIGURE 6:**
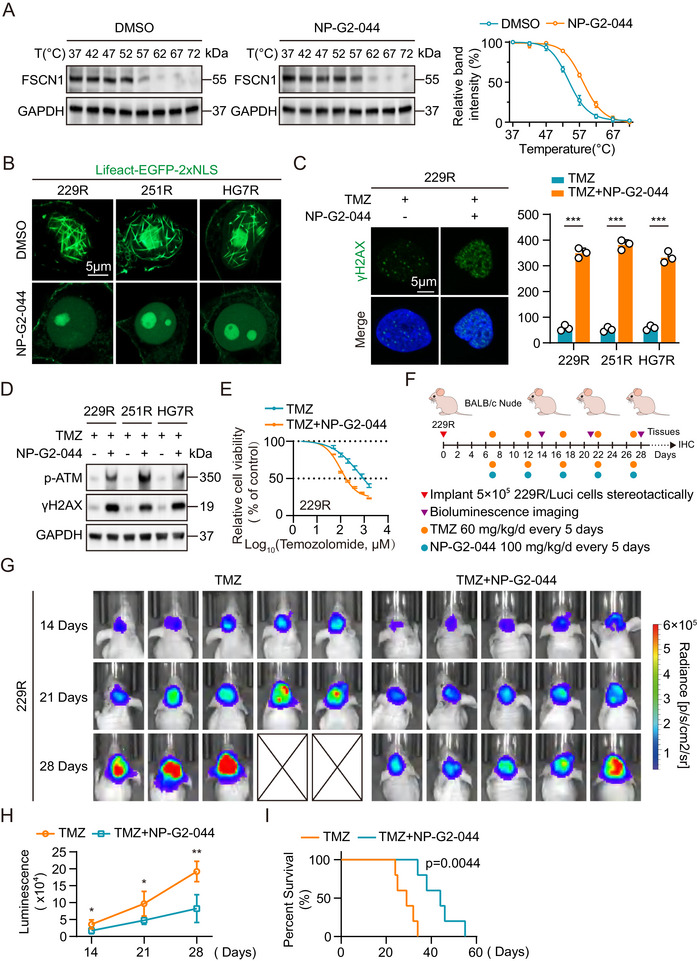
Treatment with TMZ combined with the FSCN1 inhibitor NP‐G2‐044 reverses GBM TMZ resistance both in vitro and in vivo. (A) After treatment at different temperatures ranging from 37°C to 72°C, proteins were extracted and analyzed by Western blot for FSCN1 and GAPDH. DMSO treatment served as the control group, and NP‐G2‐044 treatment as the experimental group (left). The band intensity was quantified to compare the thermal stability of FSCN1 at different temperatures (right, n = 3). (B) Immunofluorescence analysis of GBM cells treated with Lifeact‐EGFP‐2xNLS plasmids after treatment with DMSO or NP‐G2‐044. Scale bar = 5µm. (C) Immunofluorescence analysis of GBM cells stained with γH2AX antibody treated with TMZ or NP‐G2‐044. The nuclei were stained with DAPI. Quantification of γH2AX in Image J (n = 3). Scale bar = 5µm. (D) Western blot analysis of p‐ATM and γH2AX expression in TMZ‐resistant GBM cells treated with TMZ or NP‐G2‐044. E CCK‐8 assay analysis revealed the effect of TMZ‐resistant GBM cells with TMZ or NP‐G2‐044 treatment at the indicated concentrations for 72 h (n = 3). F Nude mice were orthotopically xenografted with 229R GBM cells (5 × 10^5^ cells) and treated intraperitoneally with TMZ (60 mg kg^−1^ day^−1^ per mouse) or intratumorally with NP‐G2‐044 (100 mg kg^−1^ day^−1^ per mouse) every 5 days. G Bioluminescent images of nude mice. (H) Quantification of bioluminescent imaging signal intensities in nude mice. (I) Kaplan‐Meier survival curve of nude mice is shown. For B and C, scale bars, 5 µm. Data were analyzed using Student's *t*‐test (A, E and H) and four‐parameter logistic regression (E) and Log Rank test (I). Significant results were presented as NS non‐significant, *p < 0.05, **p < 0.01, ***p < 0.001.

To explore the potential clinical application of TMZ combination therapy, we selected the FSCN1 inhibitor NP‐G2‐044, which is currently in clinical trials, primarily targeting its role in tumor invasion and metastasis [50, 51, 52]. To validate the specificity of NP‐G2‐044 for FSCN1 in GBM cells, we performed a cellular thermal shift assay (CETSA). Western blot results showed that NP‐G2‐044 significantly increased the thermal stability of FSCN1 (Figure 6A). Immunofluorescence staining revealed that in TMZ‐resistant GBM cells, NP‐G2‐044 treatment inhibited the formation of nuclear F‐actin (Figure 6B). Furthermore, NP‐G2‐044 treatment significantly increased DNA damage sites in TMZ‐resistant GBM cells under TMZ treatment (Figure 6C; Figure S7A). Western blot results showed that in TMZ‐resistant GBM cells, the combination of TMZ and NP‐G2‐044 treatment significantly elevated DNA damage marker proteins compared to TMZ treatment alone (Figure 6D). CCK8 cell viability assays demonstrated that the combination of TMZ and NP‐G2‐044 significantly inhibited the proliferative activity of TMZ‐resistant GBM cells (Figure 6E; Figure S7B). To assess the in vivo impact of NP‐G2‐044 on TMZ resistance, we established a mouse orthotopic GBM xenograft model using the TMZ‐resistant 229R cell line. Seven days after implantation, the mice received treatment with either TMZ (60 mg/kg/day) alone or TMZ combined with NP‐G2‐044 (100 mg/kg/day) every five days (Figure 6F). Bioluminescence imaging showed that combination therapy effectively restored TMZ sensitivity in TMZ‐resistant xenografts (Figure 6G). Compared to the control group, animals in the combination treatment group exhibited significantly reduced tumor volumes and extended survival (Figure 6H,I). In summary, these findings suggest that the combination of TMZ and NP‐G2‐044 may be a promising strategy to overcome TMZ resistance and enhance therapeutic efficacy.

## 3 Discussion

The standard treatment for GBM involves surgical resection followed by concurrent radiotherapy and chemotherapy. However, treatment options for recurrent GBM that is resistant to TMZ remain limited, and effective therapies to address TMZ resistance are not well established. Investigating the underlying mechanisms of TMZ resistance is crucial for developing effective combination therapies. Recent studies suggest that reorganization of the actin cytoskeleton in tumors can promote invasion, metastasis, and drug resistance [21, 53, 54]. Additionally, changes in actin occurring within the cell nucleus are critical for tumor regulation in response to various types of DNA damage [18, 19].

This study explores the connection between increased nuclear F‐actin and TMZ resistance in GBM cells. Furthermore, it was found that FSCN1, which is highly expressed in the TMZ‐resistant GBM cell nucleus, promotes TMZ resistance in GBM cells by regulating F‐actin formation. We also observed for the first time that YTHDC1 expression is significantly reduced in TMZ‐resistant GBM cells. Low YTHDC1 expression increases FSCN1 mRNA levels through m6A modification, thereby promoting TMZ resistance. Further studies revealed that FSCN1 activates CDC42 by recruiting FGD1 from the GEF family, thereby promoting the activation of the CDC42/N‐WASP/Arp2/3 pathway. After screening and validating inhibitors of key molecules in this pathway, we selected NP‐G2‐044, an inhibitor of FSCN1, which is currently being used in some clinical trials and can be safely administered orally [50, 52].

In this study, we observed the cytoskeleton in parental GBM cells and their corresponding TMZ‐resistant GBM cells and found that F‐actin formation increased in the nuclei of TMZ‐resistant GBM cells. However, current techniques for observing nuclear actin in tissue are limited due to staining challenges. Developing methods to visualize nuclear actin in tissue could provide a valuable tool for the clinical diagnosis of TMZ‐resistant GBM. Nuclear F‐actin is filamentous actin formed by the polymerization of G‐actin within the cell nucleus [39]. Previous studies have reported that F‐actin in the nucleus is involved in biological functions such as supporting nuclear shape and DNA damage repair [55]. Therefore, we further validated the relationship between nuclear F‐actin and TMZ resistance and found that when F‐actin formation in the nuclei of TMZ‐resistant GBM cells was inhibited, the cytotoxic effect of TMZ treatment on resistant cells was enhanced, and more DNA damage was produced.

After screening for relevant actin‐regulating proteins, previous reports have shown that FSCN1 exhibits biological functions in the cell nucleus and regulates F‐actin in the nucleus [27, 56]. However, the reasons for the increased expression of FSCN1 in the nucleus and the specific mechanisms underlying its regulation remain unclear. Our results indicate that FSCN1 expression is increased in the nuclei of TMZ‐resistant GBM cells and is associated with the formation of nuclear F‐actin in these cells. Gene Ontology (GO) analysis of differentially expressed genes grouped by FSCN1 expression levels in TCGA revealed significant enrichment of genes associated with DNA damage sites and DNA repair. Subsequently, we found that downregulation of nuclear FSCN1 inhibited F‐actin polymerization in the nuclei of TMZ‐resistant GBM cells, thereby reducing their DNA repair capacity. These results suggest that nuclear FSCN1 regulates nuclear actin polymerization to promote DNA damage repair and is closely associated with the development of TMZ resistance.

In eukaryotes, m6A methylation is the most prevalent mRNA modification, influencing various aspects of RNA processing, including transcription, translation, degradation, and stability [10, 57]. To investigate the factors contributing to changes in FSCN1 expression levels, based on previous research findings, we predicted several high‐confidence m6A binding sites on FSCN1 mRNA, and RIP assays confirmed that FSCN1 mRNA effectively binds to m6A antibodies. To investigate whether FSCN1 expression is regulated by m6A methyltransferases, we screened potential candidates based on sequencing data and m6A modification enzyme analysis from previous studies. YTHDC1 was the only m6A modification enzyme that was stably downregulated in TMZ‐resistant GBM cells and recurrent GBM patients. Although YTHDC1 has been associated with tumor progression in various cancers [33, 36, 38, 58, 59, 60], its role in GBM remains unexplored. Our findings indicate that YTHDC1 expression is significantly reduced in TMZ‐resistant GBM cells and recurrent GBM patients and is associated with poor prognosis and TMZ resistance. Protein‐RNA interaction assays reveal that YTHDC1 directly interacts with FSCN1 mRNA and regulates its expression. Notably, transcriptional inhibition experiments in GBM cells showed that reduced YTHDC1 levels slowed down the degradation of FSCN1 mRNA, thereby increasing its stability. In summary, our study demonstrates that reduced YTHDC1 expression in TMZ‐resistant GBM cells enhances FSCN1 mRNA stability through m6A modification, leading to FSCN1 overexpression and promoting tumor resistance.

Previous studies have shown that DSB damage sites are predominantly located within these tightly packed heterochromatic regions [24, 26]. Homologous recombination (HR) is the primary mechanism for repairing heterochromatic DSBs, but direct HR often results in repair errors. To mitigate this, heterochromatic DSBs can be relocated to the chromatin periphery for repair via the Arp2/3 complex and F‐actin, enabling more accurate HR [26]. In both fruit flies and mouse cells, this relocation process relies on nuclear actin filaments assembled by the Arp2/3 complex at repair sites, extending toward the nuclear periphery [48, 61]. Patients were grouped based on FSCN1 expression in TCGA data. Differential gene sets identified through KEGG and GSEA analysis suggested that elevated FSCN1 expression is closely associated with the actin cytoskeleton regulation pathway, and the enriched gene sets are closely related to CDC42, N‐WASP, and Arp2/3. Our findings indicate that the CDC42/N‐WASP/Arp2/3 pathway is significantly activated in TMZ‐resistant GBM cells compared to parental GBM cells and is correlated with nuclear FSCN1 expression levels. However, the biological function of FSCN1 does not directly activate CDC42. Subsequently, we performed IP assays using FSCN1 and conducted mass spectrometry analysis of the bound proteins, discovering that FGD1, as the sole GEF family molecule, possesses the ability to directly activate CDC42. Experimental results showed that increased FSCN1 activates CDC42 by recruiting more FGD1. Previous studies have reported that activated CDC42^GTP^ promotes Arp2/3‐N‐WASP complex formation by opening the specific structure of VCP on N‐WASP, thereby facilitating F‐actin polymerization. Our experimental results also confirmed this mechanism. Additionally, we observed that inhibiting FSCN1, CDC42, or Arp2/3 activity with small‐molecule inhibitors effectively disrupted nuclear F‐actin formation. This prevented DSB repair sites from relocating from heterochromatin to the chromatin periphery, thereby impairing the DNA repair process.

To fully realize the clinical potential of our findings, we investigated the therapeutic application of NP‐G2‐044, a small molecule drug that inhibits FSCN1 and is currently in clinical trials for treating tumor metastasis [50, 52]. NP‐G2‐044 is orally administered and offers promising prospects for combination therapy. In both in vitro and in vivo studies, we evaluated the combined effect of NP‐G2‐044 with TMZ on TMZ‐resistant GBM cells. NP‐G2‐044 effectively disrupted actin filament formation in resistant cells, enhancing TMZ's antitumor efficacy. In a mouse xenograft model, the combination therapy resulted in significantly smaller tumor volumes and prolonged survival compared to controls. However, the ability of NP‐G2‐044 to cross the blood‐brain barrier remains uncertain. In our experiments, mice were treated intratumorally with NP‐G2‐044, as its effectiveness via oral administration at therapeutic concentrations in the brain has yet to be established. Addressing this limitation is essential for advancing NP‐G2‐044 as a viable therapeutic option for GBM.

**FIGURE 7:**
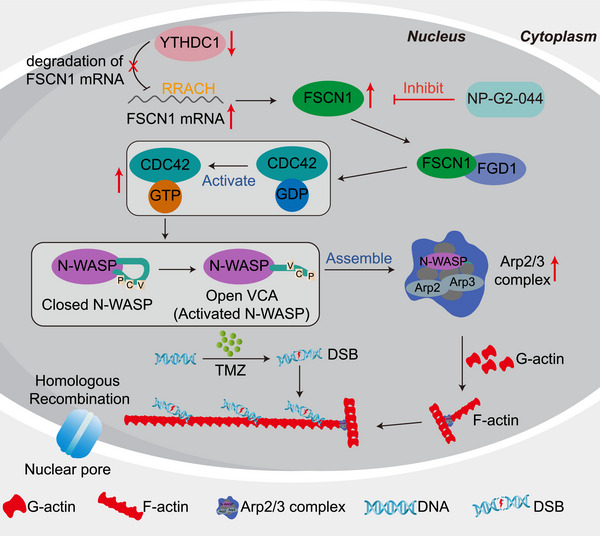
Mechanism diagram for low YTHDC1 expression upregulates FSCN1 to promote nuclear F‐actin formation and facilitate DNA breaks repair in glioblastoma.

This study highlights the critical role of nuclear F‐actin in driving TMZ resistance in GBM cells. We demonstrate that FSCN1 facilitates the polymerization of nuclear actin via the CDC42/N‐WASP/Arp2/3 pathway, thereby enhancing DNA damage repair and contributing to TMZ resistance. Additionally, reduced expression of YTHDC1 further amplifies FSCN1 levels, reinforcing this resistance mechanism. Importantly, our findings suggest that combining the FSCN1 inhibitor NP‐G2‐044 with TMZ offers a promising therapeutic approach to enhance TMZ effectiveness and overcome GBM resistance (Figure 7).

## 4 Materials and Methods

### 4.1 Cell Lines and Cell Culture

Human glioblastoma (GBM) cell lines LN229 (RRID: CVCL 0393) and U251 (RRID: CVCL 0021) were obtained from the Institute of Biochemistry and Cell Biology, Chinese Academy of Sciences (China) under catalog numbers SCSP‐502 and SCSP‐589 respectively. These cells were authenticated using STR assay (Genetic Testing Biotechnology, China). The HG7 patient‐derived line was established from primary tumor tissue of a treatment‐naive glioblastoma patient as previously described [62]. TMZ‐resistant GBM cell lines 229R, 251R and HG7R were constructed from the above cell lines as previously described, and they have been experimentally verified to be resistant to TMZ [62]. All cells were cultured in Dulbecco's modified Eagle's medium (DMEM) or DMEM/F12 with 10% fetal bovine serum (Gibco, USA) at 37°C in a humidified atmosphere with 5% CO_2_, and were tested negative for mycoplasma contamination. These cells were authenticated using STR assay (Genetic Testing Biotechnology, China). The HG7 and HG7R cells were developed to model primary human glioblastoma heterogeneity, which commercial lines cannot fully recapitulate. Their inclusion strengthens the clinical relevance of our findings by demonstrating that key results are conserved across both commercial lines and primary patient‐derived models.

### 4.2 Phalloidin Staining

Cells were seeded on confocal dishes and allowed to adhere for 24 h before fixation. First fixed them in 4% paraformaldehyde for 15 min, following fixed for 1 min in cytoskeleton buffer (10 mM MES, 150 mM NaCl, 5 mM EGTA, 5 mM glucose, 5 mM MgCl_2_, pH 6.1) containing 0.4% Triton X‐100. Subsequent occlusion of non‐specific targets with 10% goat serum (Beyotime, China). Phalloidin (Solarbio, China) was added to the dishes and incubated overnight at 4°C. After incubation, the dishes were washed three times for 5 min with 1× PBS and then incubated for 3 h at 37°C with anti‐Lamin B1 antibodies. Following another series of washes, the samples were treated with FITC‐conjugated secondary antibodies for 1 h at 37°C. After a final round of washes, the samples were visualized using a laser scanning confocal microscope (Zeiss LSM980, Germany).

### 4.3 Transfection and Imaging of Actin Overexpression Plasmids

The plasmid pEGFP‐C1 Lifeact‐EGFP‐2XNLS (Addgene, USA) was sourced from Addgene. Cells were transfected with either pEGFP‐C1 Lifeact‐EGFP‐2XNLS, and imaging was conducted 48 to 96 h post‐transfection.

### 4.4 Immunofluorescence

Cells were plated on confocal petri dishes and fixed 24 h later. Standard staining procedures were followed. Primary antibodies targeting FSCN1, YTHDC1, and γH2AX were diluted in 1% BSA in PBS and incubated overnight at 4°C. After three washes with PBS, the cells were incubated with CoraLite488‐conjugated and Cy3‐conjugated anti‐IgG antibodies (Proteintech Group, China) for 1 h at room temperature. DNA was stained with DAPI (Sigma‐Aldrich, Germany) and visualized using a laser scanning confocal microscope (Zeiss LSM980, Germany).

### 4.5 Preparation of Nuclear and Cytoplasmic Extracts

Cell cultures were washed with cold 1×PBS, scraped gently, and centrifuged at 800g for 10 min. The pellet was frozen at −80°C for 45 min, then resuspended in buffer P1 (containing 5 × 10^6^ cells in 250µL of 10mM HEPES, 0.1mM EGTA, 1mM DTT, and complete protease inhibitors). Triton X‐100 was added to a final concentration of 0.5%, and the samples were vortexed for 10 s. Nuclei were pelleted by centrifugation at 10 000g for 10 min, and the supernatant was collected as the cytoplasmic extract. The nuclear pellet was washed with buffer P1, then lysed in buffer P2 (containing 5 × 10^6^ cells in 100 µL of 20mM HEPES, 25% glycerol, 400mM NaCl, 1mM EGTA, 1mM DTT, and complete protease inhibitors) for 90 min on a rotary shaker at 4°C. Insoluble material was removed by centrifugation at 16,000g for 30 min, and the remaining supernatant was collected as the nuclear extract. The purity of the extracts was assessed by immunoblotting for α‐tubulin and histone H3.

### 4.6 Pyrenyl–Actin Assembly Assays

Nuclear extracts were dialyzed using a mini dialysis unit (Thermo Fisher Scientific, USA) against XB buffer (10mM HEPES, pH 7.7, 100mM KCl, 2mM MgCl_2_, 0.1mM CaCl_2_, 5mM EGTA, 1mM DTT) for at least 3 h. Following dialysis, the extracts were incubated with pyrene‐labeled actin (Cytoskeleton, USA), and fluorescence was measured at 407 nm with excitation at 365 nm using a Tecan Spark, controlled by SparkControl.

### 4.7 G‐Actin/F‐Actin Assay

The ratio of filamentous (F‐actin) to monomeric (G‐actin) actin was determined using a G‐actin/F‐actin in vivo assay kit (Cytoskeleton, USA) following the manufacturer's instructions. Nuclear extracts were prepared by homogenizing the samples in a lysis and F‐actin stabilization buffer. After a 10‐min incubation at 37°C, the mixture was transferred to a pre‐warmed ultracentrifuge (Beckman Coulter, USA) and centrifuged at 100,000 × g for 1 h at 37°C to separate the G‐actin (supernatant) and F‐actin (pellet) fractions. The pellet was then resuspended in depolymerizing buffer and incubated on ice for 1 h to allow actin depolymerization. Finally, all samples were mixed with 5× SDS sample buffer, heated at 95°C to denature, and analyzed by 10% SDS‐PAGE.

### 4.8 Protein Preparation and Western Blot

Total proteins were extracted from GBM cells or clinical GBM tissues using prechilled RIPA buffer containing protease and phosphatase inhibitors (Selleck.cn, China). The proteins were transferred to PVDF membranes, which were incubated overnight at 4°C with primary antibodies. The membranes were then incubated with an HRP‐conjugated secondary antibody (Zsbio Store‐bio, China) at room temperature for 1 h. Protein bands were detected using a chemiluminescence reagent (ECL) kit (Boster, China). Uncropped scans of these blots are reported in Figure S8. Antibodies for western blot are provided in Table S1.

### 4.9 Colony Formation Assay

Cells were seeded at a density of 8 × 10^2^ cells per well in a six‐well plate (Jetbiofil, China) and cultured for 11 days. Following two washes with PBS, colonies were fixed in 4% paraformaldehyde for 15 min, then stained with 0.1% crystal violet for 30 min. Colony images were captured using the ChemiDocTM MP Imaging System (Bio‐Rad, USA) and analyzed with ImageJ software for quantification.

### 4.10 Flow Cytometry Analysis

Before evaluating apoptosis, cells were treated with various compounds according to the protocol provided by the FITC Annexin‐V/propidium iodide (PI) apoptosis detection kit (BD Bioscience, USA). The proportion of apoptotic cells was determined using flow cytometry (BD Biosciences, USA). Measurements were taken 24 h after treatment with TMZ and/or LatB. In the DR‐GFP reporter system experiment, GBM cells were transfected with I‐SceI and DR‐GFP plasmids. After 48 h, cells were harvested and resuspended in 1 mL of PBS for fluorescence‐activated cell sorting (FACS) analysis. The percentage of GFP‐positive cells was determined, which allowed the assessment of DNA double‐strand break (DSB) repair efficiency. This method is commonly used to measure the repair of DSBs through homologous recombination, where the appearance of GFP‐positive cells indicates successful repair.

### 4.11 RNA Isolation and PCR

Total RNA was extracted from GBM cells or clinical tissues using TRIzol reagent (Invitrogen, USA) following the manufacturer's protocol. To isolate nuclear and cytoplasmic components, we used 0.5% NP‐40 (Solarbio, China) along with an RNAase inhibitor (Promega, USA), followed by RNA extraction with TRIzol (Sigma, USA). For cDNA synthesis, 1 µg of total RNA was used as a template, using the PrimeScript RT Reagent Kit (Takara, Japan). Real‐time quantitative PCR was performed in triplicate using SYBR Green (Takara, Japan) on a LightCycler 96 Instrument (Roche, UK). RNA levels were normalized to GAPDH, and gene expression was quantified using the 2−ΔΔCt method, with results normalized to either endogenous or exogenous reference controls. The RNA expression in tissues is normalized to the sample with the lowest expression level in the ΔΔCT processed data. Primer sequences for qRT‐PCR are provided in Table S2.

### 4.12 Comet Assay

Cells were cultured in 12‐well plates overnight and treated with TMZ (100µM) and/or LatB (1µM) for 24 h. After treatment, cells were collected and resuspended in 0.75% agarose, then placed on glass slides. Following this, the cells were lysed in lysis buffer (Trevigen, USA) for 1 h, according to the manufacturer's instructions (Beyotime, China). The slides were then subjected to electrophoresis for 30 min at 21V. DNA was stained with DAPI (Sigma, USA) and visualized using a confocal microscope.

### 4.13 Prediction of m6A Target Site

The mRNA sequence of FSCN1 was initially retrieved from NCBI. Subsequently, potential m6A binding sites were predicted using the SRAMP tool available at http://www.cuilab.cn/sramp.

### 4.14 RNA Immunoprecipitation

RIP was conducted using the Magna RIP RNA‐Binding Protein Immunoprecipitation Kit (GENE CREATE, China), following the manufacturer's instructions. Antibodies for m6A and YTHDC1 used in the assays were sourced from Abcam and Proteintech, respectively. The RNA extracted through the RIP process was then analyzed by qPCR.

### 4.15 RNA Pulldown

Using the FSCN1 mRNA sequence from NCBI, probes were designed using SnapGene software. RNA pull‐down assays were then performed with the Pierce Magnetic RNA‐Protein Pull‐Down Kit (GENE CREATE, China), following the manufacturer's guidelines. The resulting RNA‐protein complexes were analyzed by western blotting. Probe sequences were displayed in Table S3.

### 4.16 Cell Transfection

For transient overexpression experiments, the full‐length coding sequence (CDS) of the target gene was cloned into an expression vector using restriction enzymes and DNA ligase. The recombinant plasmids were transfected into cells with transfection reagents, followed by the appropriate experimental procedures. For stable overexpression, the expression vectors were introduced into 293T packaging cells to produce lentiviral particles, which were then used to infect target cells. Stable cell lines were selected with puromycin. All plasmids were obtained from Nanjing Corues Biotech. Sequences of shRNA were displayed in Table S4.

### 4.17 Immunohistochemistry

Immunohistochemistry was performed on 4µm paraffin‐embedded tissue sections using a three‐step protocol with a DAB staining kit (ZSGB‐BIO, China). First, the sections were incubated at 80°C for 15 min, followed by deparaffinization in xylene and rehydration in graded ethanol and double‐distilled water. For antigen retrieval, the slides were steamed in sodium citrate buffer at 95°C for 15 min. After washing with PBS for 3 min, the sections were incubated overnight at 4°C with primary antibodies against YTHDC1, CDC42, N‐WASP, and ARP2. Following another PBS wash, the tissues were treated with an anti‐mouse/rabbit polymer HRP label for 30 min. The staining was developed by applying the DAB chromogen solution for 0.5–1 min, resulting in a brown color development.

### 4.18 Cdc42 Activation Assay

The Cdc42 activation assay was conducted using the Cdc42 Activation Biochem Kit (BK034, Cytoskeleton) following the manufacturer's instructions. Protein was extracted from the cell lysates, and 20µL aliquots were reserved for total Cdc42 quantification. The remaining lysate was frozen in liquid nitrogen for later use. Total Cdc42 levels were measured by Western blot, and based on the results, the total Cdc42 concentration was standardized across all samples. For the Cdc42 activation assay, 200 µL of PAK‐PBD beads were incubated with the lysates at 4°C for 1 h on a rotator. After incubation, the beads were collected by centrifugation at 4000 g for 1 min at 4°C, and the supernatant was carefully discarded. The beads were washed, resuspended in 10µL of loading buffer, and boiled for 2 min. GTP‐Cdc42 levels were then analyzed by Western blot.

### 4.19 Cellular Thermal Shift Assay (CETSA)

Cells in the logarithmic growth phase were washed with PBS, digested, counted, and resuspended in pre‐warmed culture medium. Then, 10 µM NP‐G2‐044 or an equal volume of DMSO was added, and the cells were incubated at 37°C for 1 h. Subsequently, cells from each treatment group were heated in a metal bath at 37°C, 42°C, 47°C, 52°C, 57°C, 62°C, 67°C, and 72°C for 3 min, followed by immediate cooling at room temperature or 4°C for 3 min. Protein extraction was performed, followed by Western blot analysis. The density of the soluble FSCN1 bands at each temperature point was analyzed using ImageJ software. The signal at 37°C for each treatment group was normalized (set as 1.0) and nonlinear regression analysis was performed using the four‐parameter logistic regression or Boltzmann equation in GraphPad Prism. Three independent biological replicates were performed for each condition.

### 4.20 Molecular Medicine

All small molecule drugs were purchased from MedChemExpress and dosages were administered according to the manufacturer's instructions and references in the recommended list. Molecule drugs are provided in Table S5.

### 4.21 Xenograft Model In Vivo

Four‐week‐old female athymic BALB/c nude mice were purchased from GemPharmatech Co., Ltd. (China). Each mouse received a stereotactic injection of 5 × 10⁵ GBM cells (229R) into the brain. Upon meeting the enrollment criteria, the mice were randomized into groups using a 1:1 computer‐generated randomization scheme. Throughout the study, the drug administration personnel, imaging/pathology evaluation personnel, and statisticians were blinded to the group allocation. Each group consisted of n = 5 animals. Exclusion criteria included failure to form tumors, death prior to randomization, or occurrence of unexpected events unrelated to the intervention after randomization. The survival endpoint was defined as the time from randomization to meeting humane endpoint criteria (e.g., weight loss ≥20%, increased lethargy/neurodeficits, or bioluminescence imaging confirming excessive intracranial tumor burden) or natural death. After surgery, the mice were treated for 3 weeks with TMZ (60 mg/kg/day) and/or NP‐G2‐044 (100 mg/kg/day). In the TMZ and NP‐G2‐044 treatment regimen, a staggered dosing schedule was employed. According to previous reports, NP‐G2‐044 did not show significant hepatic or renal toxicity. In our experiment, NP‐G2‐044 was administered via stereotactic intracranial injection, TMZ was administered intraperitoneally 3 h after NP‐G2‐044 administration to ensure overlap in the plasma drug concentrations while minimizing potential toxicity from peak concentrations. Intracranial tumor growth was monitored using bioluminescence imaging. After the treatment period, the mice were euthanized, and brain tissues were collected. These tissues were paraffin‐embedded and sectioned at 4 µm thickness for immunohistochemical analysis. All animal experiment protocols were approved by the Animal Ethics Committee of the University of Science and Technology of China (approval No. 2022‐N(A)‐104) and were conducted in accordance with the University's Laboratory Animal Use Guidelines.

### 4.22 Human Glioma Specimens

All pathologically identified glioma samples used in this study were obtained from patients in the Department of Neurosurgery at The First Affiliated Hospital of the University of Science and Technology of China, with written informed consent. This study was approved by the Medical Research Ethics Committee of The First Affiliated Hospital of USTC and conducted in accordance with all relevant ethical regulations (approval No. 2024KY‐578). The basic characteristics of participants are shown in Table S8.

### 4.23 Bioinformatics Analysis

Gene expression, gene correlation, and survival data for this study were sourced from the TCGA (https://www.cancer.gov/ccg/research/genome‐sequencing/tcga) and CGGA (http://www.cgga.org.cn/) databases. Data integration and analysis were performed using R. Transcriptome‐related analysis data were retrieved from the GEO database (https://www.ncbi.nlm.nih.gov/geo/). Prediction of m6A sites were performed in SRAMP (http://www.cuilab.cn/m6asiteapp/old). Prediction of m6A sequences are provided in Table S6. The data from the FSCN1 IP followed by liquid chromatography‐mass spectrometry (LC‐MS) are presented in Table S7. Differential gene expression analysis was conducted with R, followed by annotation and enrichment analysis of the identified genes.

### 4.24 Data Availability

The transcriptome expression profile was obtained from TCGA (http://cancergenome.nih.gov/) and CGGA (http://www.cgga.org.cn/) database. The primary and recurrent patients’ mRNA‐seq data were obtained from the GSE62153 dataset. The mRNA expression profiling of parental and TMZ‐resistant glioma cells was obtained from the GSE113510 dataset and the GSE100736 dataset.

### 4.25 Statistical Analysis

Statistical comparisons between groups were performed using Student's *t*‐test for two groups, and one‐way analysis of variance (ANOVA) for comparisons involving three or more groups. Survival distributions were illustrated using Kaplan‐Meier curves, with statistical significance assessed by the log‐rank test. Univariate and multivariate Cox regression analyses were conducted to further explore survival data. Correlations between variables were evaluated using the Pearson correlation coefficient. Gene Ontology (GO) and Kyoto Encyclopedia of Genes and Genomes (KEGG) pathway analyses were carried out via the DAVID platform (http://david.abcc.ncifcrf.gov/home.jsp). Statistically significant gene sets were visualized in Cytoscape, and Gene Set Enrichment Analysis (GSEA) was used to explore biological processes. Heatmaps were generated using Gene Cluster 3.0 and Gene Tree View software. All data are presented as mean ± SD, with *p*‐values < 0.05 considered statistically significant. The immunofluorescence images were quantified using ImageJ‐win64 (National Institutes of Health, Germany). Statistical analyses were performed using GraphPad software version 9.5 (GraphPad Software, USA).

## Author Contributions

C.N. conceived the platform, gathered funding, and provided guidance with experimental design. P.W. gathered funding and provided guidance with experimental design and led the project. M.Y. and W.N. co‐wrote the paper. Y.W., P.C., M.M., X.Z., and B.X. designed the animal experiments and analyzed the results. M.Y., W.N., S.H. and Y.W. contributed to data analysis and provided advice on this work. All authors contributed to data interpretation, discussion of results, and commented on the manuscript.

## Funding

This study was supported by the National Natural Science Foundation of China (No: 82573367, NO: 82202868, No: 82273281), the China Postdoctoral Science Foundation (NO: 2022TQ0326, NO:2023M733391), the Natural Science Foundation of Anhui Province (No: 2208085QH251), Special Fund Project for Guiding Local Science and Technology Development by the Central Government (No: 2019b07030001), the Fundamental Research Funds for the Central Universities (No: WK9110000145), 2022 Anhui Province Postdoctoral Researchers' Scientific Research Activity Funding Project (No: 2022B580), Open Project of Anhui Provincial Key Laboratory of Tumor Evolution and Intelligent Diagnosis and Treatment (No: KFKT202401).

## Conflicts of Interest

The authors declare no conflicts of interest.

## Supporting information




**Supporting File 1**: advs73491‐sup‐0001‐SuppMat.docx.

**Supporting File 2**: advs73491‐sup‐0002‐Table S1.xlsx.

**Supporting File 3**: advs73491‐sup‐0003‐Table S2.xlsx.

**Supporting File 4**: advs73491‐sup‐0004‐Table S3.xlsx.

**Supporting File 5**: advs73491‐sup‐0005‐Table S4.xlsx.

**Supporting File 6**: advs73491‐sup‐0006‐Table S5.xlsx.

**Supporting File 7**: advs73491‐sup‐0007‐Table S6.xlsx.

**Supporting File 8**: advs73491‐sup‐0008‐Table S7.xlsx.

**Supporting File 9**: advs73491‐sup‐0009‐Table S8.xlsx.

## Data Availability

The data that support the findings of this study are available on request from the corresponding author. The data are not publicly available due to privacy or ethical restrictions.
