# Supplementary material for: Low YTHDC1 Expression Upregulates FSCN1 to Promote Nuclear F‐Actin Formation and Facilitate Double‐strand DNA Breaks Repair in TMZ‐Resistant Glioblastoma
Source: Adv Sci (Weinh). 2025 Dec 27;13(13):e13632. doi: 10.1002/advs.202513632 (PMC12955858; doi:10.1002/advs.202513632)
Supplement: Supplementary file 1 — Supporting File 1: advs73491‐sup‐0001‐SuppMat.docx. [file ADVS-13-e13632-s008.docx]

**
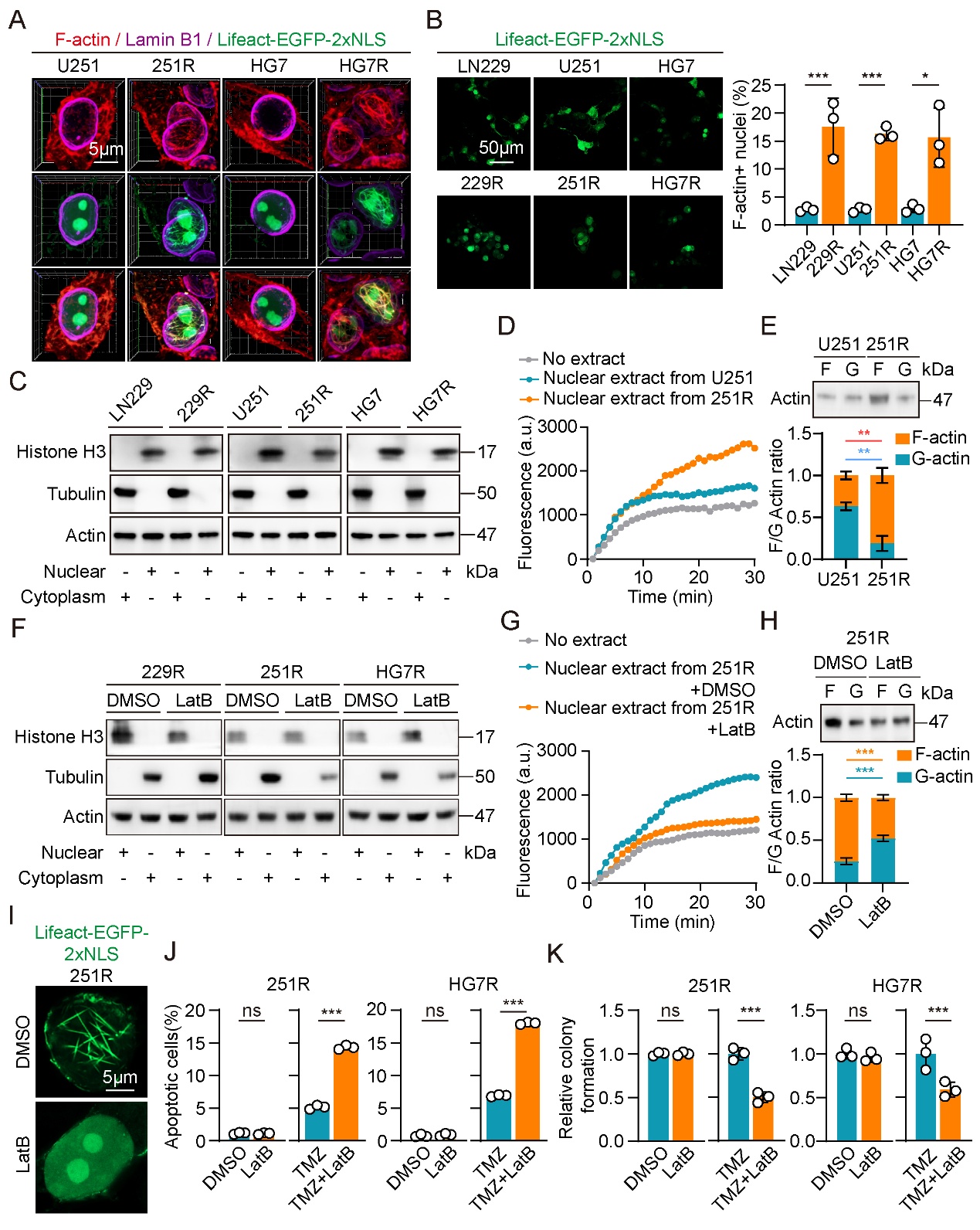
**

**Supplementary Figure 1. Nuclear F-actin increase and promote TMZ resistance in GBM cells**

**A** Immunofluorescence analysis of GBM cells stained with phalloidin, anti-Lamin B1 antibody and Lifeact-EGFP-2xNLS plasmids. Scale bar = 5μm. **B** Immunofluorescence analysis of GBM cells treated with Lifeact-EGFP-2xNLS plasmids and statistical analysis of the proportion of F-actin positivity in the cell nucleus (n = 3), Scale bar = 50μm. **C** Western Blots of nuclear and cytosolic extracts used in the above experiment from cells. The loaded volume of nuclear to cytoplasmic extract is 5:1. **D** Normalized timecourse of pyrene-labelled actin assembly in the U251 and 251R nuclear extracts. **E** Western Blot (top) and analysis of globular (G-) and filamentous (F-) actin indicate the rate of actin polymerization in TMZ-resistant cells (n = 3). **F** Western blots of nuclear and cytosolic extracts used in the above experiment from cells with DMSO or LatB treatment. The loaded volume of nuclear to cytoplasmic extract is 5:1. **G** Normalized timecourse of pyrene-labelled actin assembly in the TMZ-resistant GBM cells nuclear extracts. **H** Western Blot (top) and analysis of globular (G-) and filamentous (F-) actin indicate the rate of actin polymerization in TMZ-resistant cells after DMSO or LatB treatment (n = 3). **I** Immunofluorescence analysis of GBM cells treated with Lifeact-EGFP-2xNLS plasmids after treatment with DMSO or LatB. Scale bar = 5μm. **J** Flow cytometric analysis revealed the effect of F-actin suppressed on the apoptosis of TMZ-resistant cells with or without TMZ treatment (n = 3). **K** Colony formation assay detected the effect of F-actin suppressed on the growth of TMZ-resistant cells with or without TMZ treatment in a 6-well dish (800 cells per well) for 11 days (n = 3). For A, I, scale bars, 5µm. For B, scale bars, 50µm. Data were analyzed using Student’s t-test (B, E, H, J and K). Significant results were presented as NS non-significant, *P < 0.05, **P < 0.01, ***P < 0.001.

**
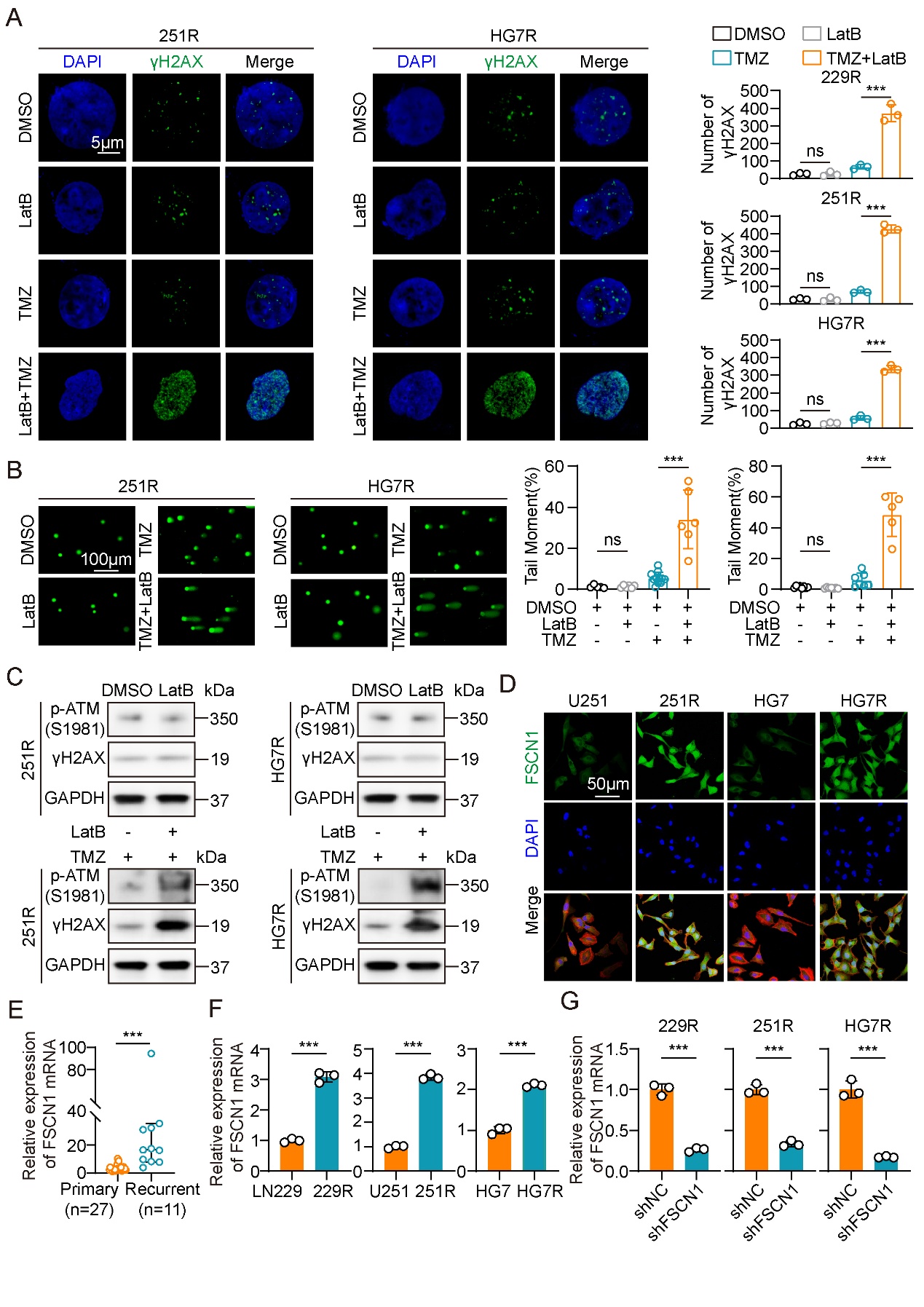
**

**Supplementary Figure 2. FSCN1 regulates nuclear F-actin formation to promote DNA damage repair**

**A** Immunofluorescence analysis of γH2AX in 251R and HG7R treated with DMSO, LatB, TMZ or TMZ + LatB. The nuclei were stained with DAPI. Quantification of γH2AX in Image J (n = 3). Scale bar = 5μm. **B** Comet assays measuring the DNA damage degree in 251R and HG7R treated with DMSO, LatB, TMZ or TMZ + LatB. Scale bar = 100μm. Comet tails were measured by image j. **C** Western blot analysis of p-ATM and γH2AX expression in 251R and HG7R treated with DMSO, LatB, TMZ or TMZ + LatB. **D** Immunofluorescence analysis of FSCN1 in parental and TMZ-resistant GBM cells. Scale bar = 50μm. **E** Relative expression of FSCN1 in primary (n = 27) and recurrent (n = 11) GBM sample tissues. **F** Relative expression of FSCN1 mRNA in parental and TMZ-resistant GBM cells (n = 3). **G** Relative expression of FSCN1 mRNA in FSCN1 knockdown TMZ-resistant GBM cells (n = 3). For A, scale bars, 5µm. For D, scale bars, 50µm. For B, scale bars, 100µm. Data were analyzed using Student’s t-test (A, B, E, F, and G). Significant results were presented as NS non-significant, *P < 0.05, **P < 0.01, ***P < 0.001.

**
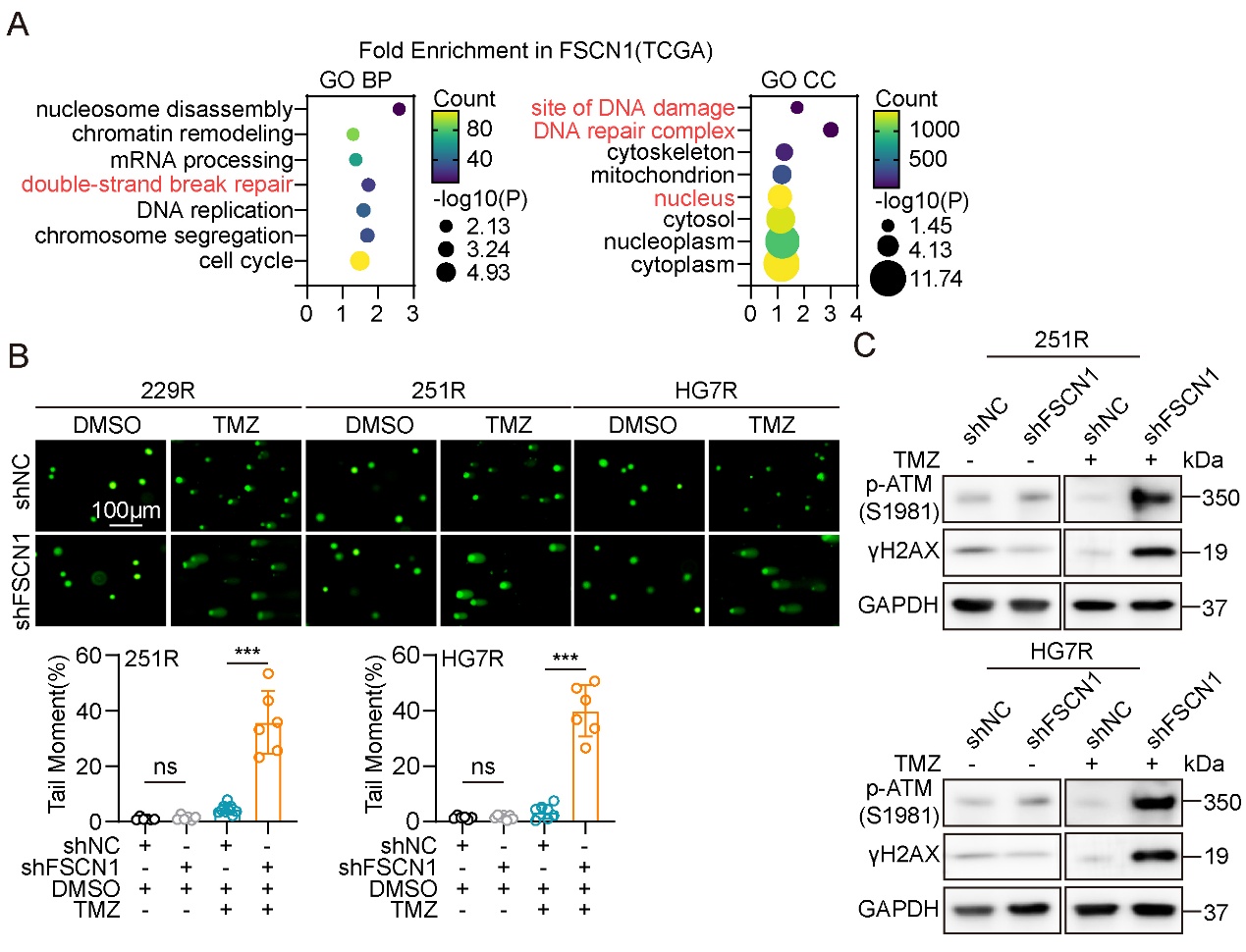
**

**Supplementary Figure 3. FSCN1 regulates nuclear F-actin formation to promote DNA damage repair**

**A** Association between FSCN1 expression and functional gene sets in TCGA dataset. **B** Comet assays measuring the DNA damage degree in FSCN1 knockdown 251R and HG7R cells treated with DMSO or TMZ. Scale bar = 100μm. Comet tails were measured by image j. **C** Western blot analysis of p-ATM and γH2AX expression in FSCN1 knockdown 251R and HG7R cells treated with or without TMZ. For B, scale bars, 100µm. Data were analyzed using Student’s t-test (B). Significant results were presented as NS non-significant, *P < 0.05, **P < 0.01, ***P < 0.001.

**
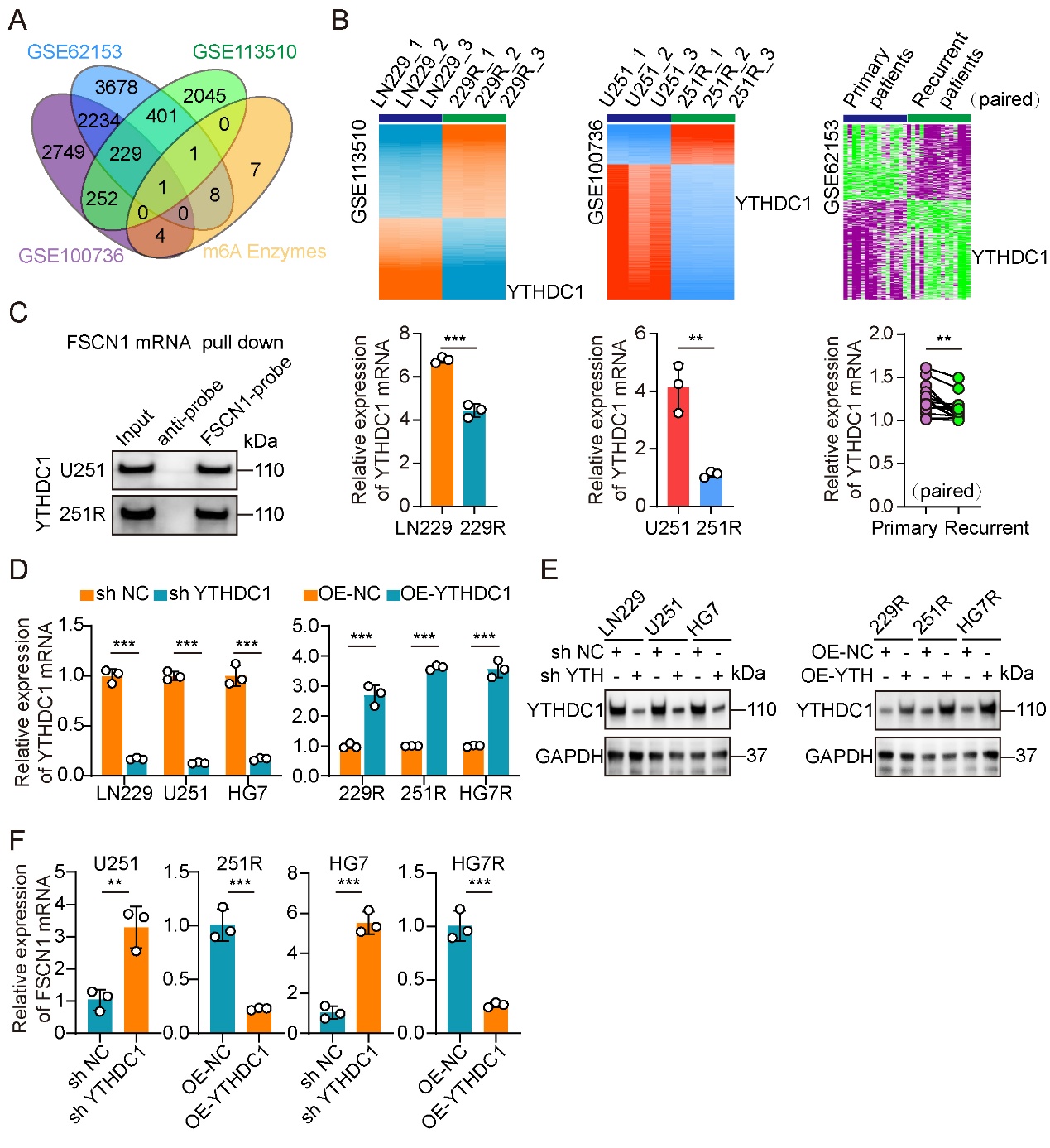
**

**Supplementary Figure 4. YTHDC1 regulates FSCN1 expression through m6A modification**

**A** Venn diagrams showing the overlaps between GSE62153, GSE113510, GSE100736 and m6A Enzymes datasets. **B** Heatmap and relative expression of YTHDC1 mRNA in GSE62153, GSE113510 and GSE100736 datasets. **C** Western blot analysis of YTHDC1 after pulldown by FSCN1 mRNA probes in parental and TMZ-resistant GBM cells. **D** Relative expression of YTHDC1 mRNA in YTHDC1 knockdown parental and YTHDC1 overexpression TMZ-resistant GBM cells (n = 3). **E** Western blot analysis of YTHDC1 in YTHDC1 knockdown parental and YTHDC1 overexpression TMZ-resistant GBM cells. **F** Relative expression of FSCN1 mRNA in YTHDC1 knockdown U251 and YTHDC1 overexpression 251R (n = 3). Data were analyzed using Student’s t-test (B, D and F). Significant results were presented as NS non-significant, *P < 0.05, **P < 0.01, ***P < 0.001.

**
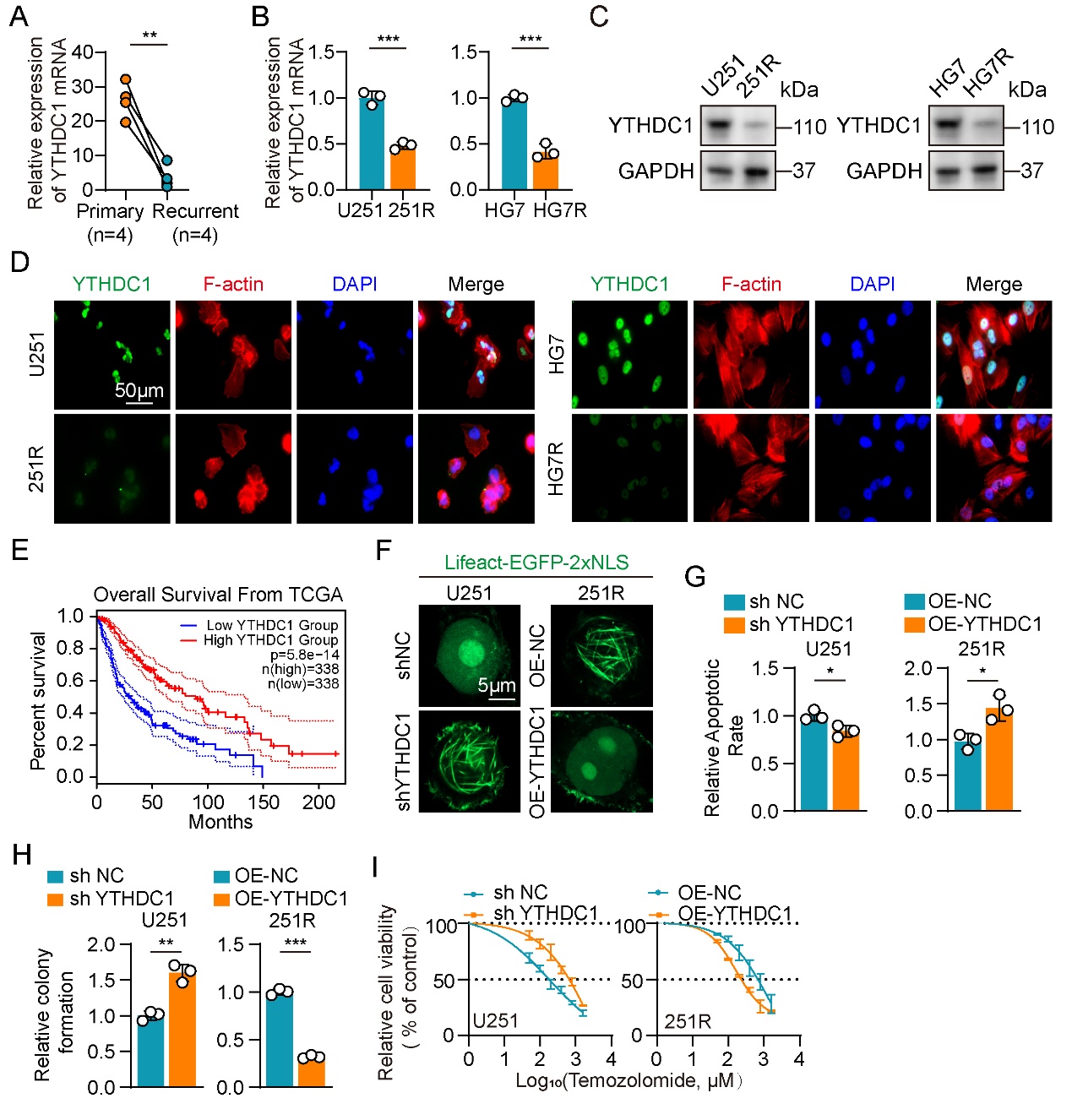
**

**Supplementary Figure 5. YTHDC1 downregulation in TMZ-resistant GBM cells and recurrent patients correlates with TMZ resistance**

**A** Relative expression of YTHDC1 mRNA in paired primary and recurrent tissues from clinical patients (n = 4). **B** Relative expression of YTHDC1 mRNA in parental and TMZ-resistant GBM cells (n = 3). **C** Western blot analysis of YTHDC1 expression in parental and TMZ-resistant GBM cells. **D** Immunofluorescence analysis of YTHDC1 expression in parental and TMZ-resistant GBM cells. The nuclei were stained with DAPI. Scale bar = 50μm. **E** Kaplan-Meier curves of OS in GBM patients of YTHDC1 expression from TCGA. **F** Immunofluorescence analysis of YTHDC1 expression in GBM cells treated with Lifeact-EGFP-2xNLS plasmids. Scale bar = 5μm. **G** Flow cytometric analysis revealed the apoptosis of YTHDC1 knockdown U251 and YTHDC1 overexpression 251R cells with TMZ treatment (n = 3). **H** Colony formation assay detected the growth of YTHDC1 knockdown U251 and YTHDC1 overexpression 251R cells with TMZ treatment in a 6-well dish (800 cells per well) for 11 days (n = 3). **I** CCK-8 assay analysis revealed the effect of TMZ-resistant GBM cells with OE-YTHFC1 or parental GBM cell with shYTHDC1 at the indicated concentrations for 72 h (n = 3). For F, scale bars, 5µm. For D, scale bars, 50µm. Data were analyzed using Student’s t-test (A, B, G and H) and Log Rank test (E) and four-parameter logistic regression (I). Significant results were presented as NS non-significant, *P < 0.05, **P < 0.01, ***P < 0.001.

**
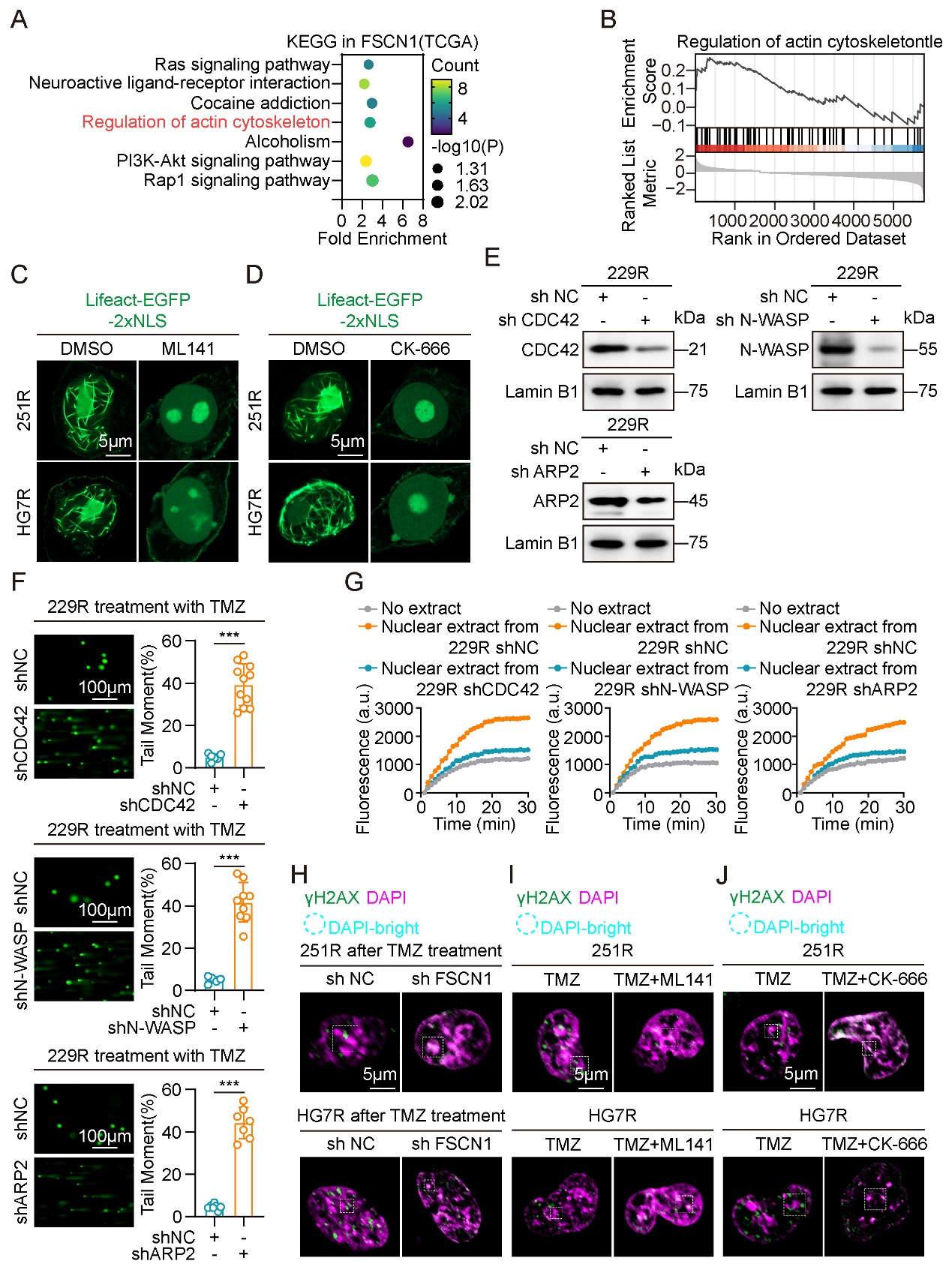
**

**Supplementary Figure 6. FSCN1 activates the CDC42/N-WASP/Arp2/3 axis by recruiting FGD1**

**A** Association between FSCN1 expression and KEGG pathway in TCGA dataset. **B** GSEA of the regulation of actin cytoskeleton signaling pathway was performed in high expression and low expression of FSCN1 from TCGA. **C** Immunofluorescence analysis of 251R and HG7R treated with Lifeact-EGFP-2xNLS plasmids after treatment with DMSO or ML141(10 μM, 37 °C, 1 hour). Scale bar = 5μm. **D** Immunofluorescence analysis of 251R and HG7R treated with Lifeact-EGFP-2xNLS plasmids after treatment with DMSO or CK-666 (100 μM, 37 °C, 6 hour). Scale bar = 5μm. **E** Western blot analysis of CDC42 in CDC42 knockdown 229R cells, N-WASP in N-WASP knockdown 229R cells, ARP2 in ARP2 knockdown 229R cells. **F** Comet assays measuring the DNA damage degree in 229R shCDC42, 229R shN-WASP and 229R shARP2 cells treated with TMZ. Scale bar = 100μm. Comet tails were measured by image j. **G** Normalized timecourse of pyrene-labelled actin assembly in the 229R shCDC42, 229R shN-WASP and 229R shARP2 cells’ nuclear extracts. **H** Immunofluorescence of γH2AX foci in FSCN1 knockdown 251R and HG7R with TMZ treatment show γH2AX foci in DAPIbright heterochromatin. Scale bar = 5μm. **I** Immunofluorescence of γH2AX foci in 251R and HG7R with TMZ or TMZ + ML141 (10 μM, 37 °C, 1 hour) treatment show γH2AX foci in DAPIbright heterochromatin. Scale bar = 5μm. **J** Immunofluorescence of γH2AX foci in 251R and HG7R with TMZ or TMZ + CK-666 (100 μM, 37 °C, 6 hour) treatment show γH2AX foci in DAPIbright heterochromatin. Scale bar = 5μm. For C, D, H, I and J, scale bars, 5µm. For F, scale bars, 100µm. Data were analyzed using Student’s t-test (F). Significant results were presented as NS non-significant, *P < 0.05, **P < 0.01, ***P < 0.001.

**
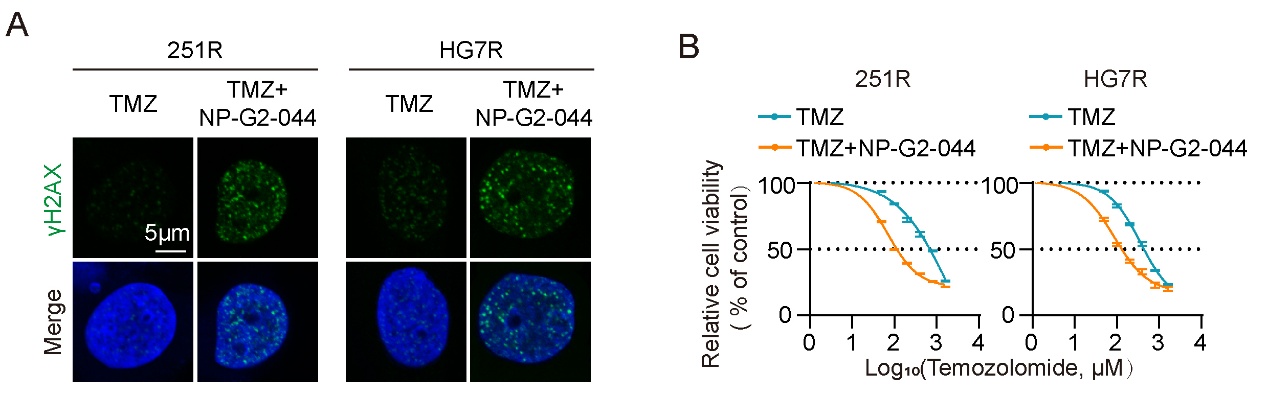
**

**Supplementary Figure 7. Treatment with TMZ combined with the FSCN1 inhibitor NP-G2-044 reverses GBM TMZ resistance both in vitro and in vivo**

**A** Immunofluorescence analysis of 251R and HG7R stained with γH2AX antibody treated with TMZ or NP-G2-044. The nuclei were stained with DAPI. Quantification of γH2AX in Image J. Scale bar = 5μm. **B** CCK-8 assay analysis revealed the effect of TMZ-resistant GBM cells with TMZ or NP-G2-044 treatment at the indicated concentrations for 72 h (n = 3). For A, scale bars, 5µm. Data were analyzed using our-parameter logistic regression (B). Significant results were presented as NS non-significant, *P < 0.05, **P < 0.01, ***P < 0.001.
